# Routes to Pt Derivatives of High‐Valent Sulfur Oxofluorides S(═O)_2_F, S(═O)F_2_, and S(═O)F_3_ by Fluorination and Oxygenation

**DOI:** 10.1002/anie.202503153

**Published:** 2025-04-07

**Authors:** Ruben Jaeger, Ouchan He, Stefan Sander, Dilcan Dirican, Mike Ahrens, Thomas Braun

**Affiliations:** ^1^ Department of Chemistry Humboldt‐Universität zu Berlin Brook‐Taylor‐Straße 2 12489 Berlin Germany

**Keywords:** Fluorido complexes, Fluorination, Platinum, S─F activation, Sulfur fluorides

## Abstract

Metal derivatives of high‐valent sulfur fluorides and oxofluorides can provid e fluorinated building blocks for materials science and bioactive compounds, but so far, such building blocks are elusive. The paper describes routes to access remarkable metal derivatives of S(═O)_2_F, S(═O)F_2_, and S(═O)F_3_ by oxygenation or fluorination steps. The Pt(II) fluorido complex *trans*‐[Pt(F)(SOF)(PCy_3_)_2_] (**2**) reacts with the Davis reagent (3‐phenyl‐2‐(phenylsulfonyl)‐1,2‐oxaziridine) to yield the sulfuryl fluorido complex *trans*‐[Pt(F)(SO_2_F)(PCy_3_)_2_] (**4**). Notably, the electrophilic fluorinating agent NFSI (*N*‐fluorobenzenesulfonimide) reacts with **2** to form *trans*‐[Pt(F)(SOF_2_)(PCy_3_)_2_][NFSO_2_Ph] (**5a**). By nucleophilic fluorination with TMAF (Me_4_NF) it is possible to fluorinate the sulfur center once more to give the complex *trans*‐[Pt(F)(SOF_3_)(PCy_3_)_2_] (**6**) bearing an unprecedented SOF_3_ ligand. Above 283 K, complex **6** shows a decomposition of the SOF_3_ moiety to form *trans*‐[Pt(F)_2_(PCy_3_)_2_] (**7**) and SOF_2_. The described complexes could represent a previously unknown class of transfer reagents for high‐valent sulfur fluoride units.

## Graphical abstract

This study demonstrates the controlled functionalization of a SOF ligand at a platinum center, achieving the selective formation of SO_2_F and SOF_2_ complexes by fluorination or oxygenation. Further fluorination of the SOF_2_ ligand resulted in an unprecedented SOF_3_ metal derivative, which is a unique example of a high‐valent fluorinated sulfur atom coordinated to a metal center.

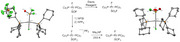

Fluorinated building blocks hold great importance in fields such as materials sciences, catalysis, medicine, and biochemistry.^[^ ^1^, ^2^, ^3^ ^]^ Therefore, the development of routes to access fluorinated compounds continues to be of high interest. Sulfur fluorides such as SF_4_ and its derivatives, as well as in the recent years also SF_6_ can be applied as fluorinating agents, whereas the SF_5_ group exhibits a high chemical stability, electronegativity, and lipophilicity.^[^ ^4^, ^5^, ^6^, ^7^, ^8^, ^9^, ^10^, ^11^, ^12^, ^13^, ^14^, ^15^, ^16^, ^17^, ^18^, ^19^, ^20^, ^21^, ^22^, ^23^ ^]^ The synthesis of metal derivatives of sulfur fluorides remains, however, very challenging. SF_4_ is renowned for its ability to add by oxidative addition to Rh, Ir, and Pt centers under the formation of SF_3_ and fluorido ligands.^[^ ^24^, ^25^, ^26^, ^27^, ^28^, ^29^, ^30^, ^31^, ^32^ ^]^ Thus, SF_4_ exhibits a notable reactivity toward Pt(0) phosphine complexes such as [Pt(PR_3_)_2_] (R═Cy, *i*Pr) resulting in the formation of *trans*‐[Pt(F)(SF_3_)(PR_3_)_2_]. The platinum complex *trans*‐[Pt(F)(SF_3_)(PCy_3_)_2_] can be used as deoxyfluorinating agent for the fluorination of ethanol to give *trans*‐[Pt(F)(SOF)(PCy_3_)_2_] (**2**), fluoroethane, and HF.^[^ ^32^, ^33^ ^]^ Reports on the reactions of SF_5_Cl with Rh(I) and Ir(I) precursors [M(X)(CO)(PEt_3_)_2_] (M═Rh, Ir; X═Cl, Br, I, NCO, and NCS) and *trans*‐[Rh(Cl)(CO)(IMes)_2_] resulted in the generation of SF_3_ complexes or led to the formation of the SF_5_ anion, which exhibits non‐nucleophilic behavior.^[^ ^27^, ^28^, ^34^, ^35^, ^36^, ^37^, ^38^, ^39^, ^40^ ^]^ Contrarily, the reactions of SO_2_F_2_ (sulfuryl fluoride) or SO_2_FCl (sulfuryl chloride fluoride) with various transition metal complexes often resulted solely in the halogenation of the metal center, and no SO_2_F or SO_2_Cl complexes were observed.^[^ ^41^, ^42^, ^43^, ^44^, ^45^, ^46^, ^47^ ^]^ However, the only exception is [Re(SO_2_F)(CO)_5_], which is accessible from [Re(SO_2_)(CO)_5_]^+^ and SO_2_F^−^.^[^ ^48^ ^]^ It remained unclear whether the SO_2_F unit in the ligand sphere of {Re(CO)_5_} was formed by fluorination of the SO_2_ ligand or by replacement of the SO_2_ ligand by the anion. Complexes bearing SF_2_, SF_3_, and SOF ligands have been previously characterized in the literature.^[^ ^24^, ^25^, ^26^, ^27^, ^28^, ^29^, ^30^, ^31^, ^32^, ^33^, ^49^ ^]^ However, there are no reports of transition metal complexes featuring a high‐valent polyfluorinated sulfur atom, and the preparation of metal SF_5_ compounds is so far elusive.^[^ ^25^, ^50^ ^]^ Note that a report exists concerning a Pt complex bearing a SF_5_ ligand, but the findings proved to be incorrect.^[^ ^51^ ^]^ In this study, we present synthetic pathways to access remarkable metal derivatives of SO_2_F, SOF_2_, and SOF_3_ ligands. The Pt(II) fluorido complexes are prepared by fluorination or oxygenation reactions of sulfur‐containing ligands.

A reaction of the platinum(0) complex [Pt(PCy_3_)_2_] (**1**) with a solution of SOF_2_ in THF (0.01 M) at room temperature led to the formation of the platinum(II) fluorido complex *trans*‐[Pt(F)(SOF)(PCy_3_)_2_] (**2**) after 3 h, which has been identified before by hydrolysis of *trans*‐[Pt(F)(SF_3_)(PR_3_)_2_] (Scheme 1).^[^ ^32^ ^]^ X‐ray crystallography of **2** revealed that the S1─F2 bond length (1.637(2)) is longer and the S1─O2A bond length (1.407(2)) is similar when compared to the S─F and S─O bond distances for the SO_2_F and SOF_2_ moieties in complexes **4** and **5b** (see below) (Figure 1).^[^ ^52^ ^]^

**Scheme 1:**
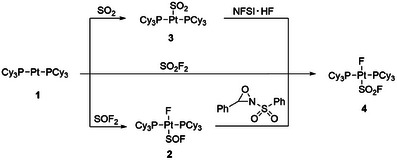
Reaction pathways to form *trans‐*[Pt(F)(SOF)(PCy_3_)_2_] (**2**), [Pt(SO_2_)(PCy_3_)_2_] (**3**), and *trans‐*[Pt(F)(SO_2_F)(PCy_3_)_2_] (**4**) from [Pt(PCy_3_)_2_] (1).

**Figure 1:**
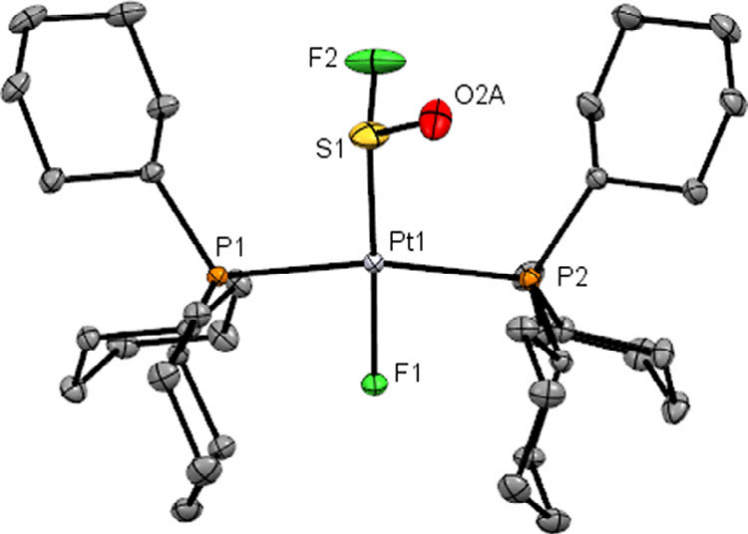
Structure of **2**.^[^ ^32^, ^53^ ^]^ Thermal ellipsoids are drawn at 50 % probability level. Carbon‐bound hydrogen atoms were omitted for clarity. Selected distances [Å] and angles [°]: Pt1─S1 2.1958(7), Pt1─F1 2.047(1), Pt1─P1 2.3359(5), Pt1─P2 2.3383(6), S1─F2 1.637(2), S1─O2A 1.407(2); F1─Pt1─S1 177.26(4), and F1─Pt1─P1 84.38(4).

Remarkably, treatment of **2** with the Davis reagent (3‐phenyl‐2‐(phenylsulfonyl)‐1,2‐oxaziridine) at room temperature in THF gave the fluorosulfonyl complex *trans*‐[Pt(F)(SO_2_F)(PCy_3_)_2_] (**4**) by oxygenation (Scheme 1). An alternative approach to synthesize **4** consists of a reaction of **1** with SO_2_F_2_, which represents an unprecedented S─F bond oxidative addition of a S(VI) fluoride. Note that **1** reacts with SF_6_ to yield the SF_3_ complex *trans*‐[Pt(F)(SF_3_)(PCy_3_)_2_], rather than an elusive SF_5_ complex.^[^ ^32^ ^]^ The formation of **4** has also been observed by treatment of [Pt(SO_2_)(PCy_3_)_2_] (**3**) with NFSI (*N*‐fluorobenzenesulfonimide) in the presence of HF (Scheme 1). Note that NFSI does not react with SO_2_. However, it is reported that arenesulfonyl fluorides can be obtained using mercaptans, thioethers, and disulfides as substrates via extensive oxidative fluorination with NFSI.^[^ ^54^, ^55^, ^56^, ^57^, ^58^, ^59^, ^60^ ^]^ Notably, the reaction pathway using the Davis reagent is substantially more selective than the other two reactions mentioned above. Fluorination or oxygenation of transition metal complexes at a sulfur atom coordinated to the metal center is extraordinary.^[^ ^61^ ^]^

The ^19^F NMR spectrum of **4** shows a signal for the SO_2_F ligand and one for the fluorido ligand bound at the platinum center which integrate 1:1. The signal at *δ* = 126 ppm with ^195^Pt satellites corresponds to the sulfur bound fluoride which shows a doublet of triplets splitting pattern with ^3^ *J*_F,P_ = 4 Hz, ^3^ *J*_F,F_ = 43 Hz, and ^2^ *J*_F,Pt_ = 980 Hz. A doublet of triplets at *δ* = −310 ppm with coupling constants of ^2^ *J*_F,P_ = 22 Hz and ^3^ *J*_F,F_ = 43 Hz reveals the presence of the metal bound fluorido ligand.^[^ ^24^, ^32^, ^33^, ^62^, ^63^, ^64^, ^65^, ^66^, ^67^, ^68^, ^69^, ^70^ ^]^ The ^31^P{^1^H} NMR spectrum of **4** shows one signal at *δ* = 33.8 ppm as a doublet of doublets with ^2^ *J*_P,F_ = 22 Hz, ^3^ *J*_P,F_ = 4 Hz, and ^1^ *J*_P,Pt_ = 2500 Hz. The magnitude of the latter is in good accordance to known phosphorus‐platinum(II) couplings.^[^ ^32^, ^33^, ^62^, ^70^, ^71^, ^72^ ^]^ Colorless crystals suitable for single X‐ray crystallography of **4** were obtained by recrystallization from THF (Figure 2). The structure of **4** reveals a slightly distorted square‐planar coordination geometry at the metal center in which the fluorosulfonyl ligand is located in a *trans* position to the metal fluoride. The Pt1─S1 as well as Pt1─F1 bond lengths are in good agreement with the ones in complex *trans*‐[Pt(F)(SOF)(PCy_3_)_2_] (**2**). The sulfur atom exhibits a distorted tetrahedral arrangement of the Pt1, O1, O2, and F1 atoms.

**Figure 2:**
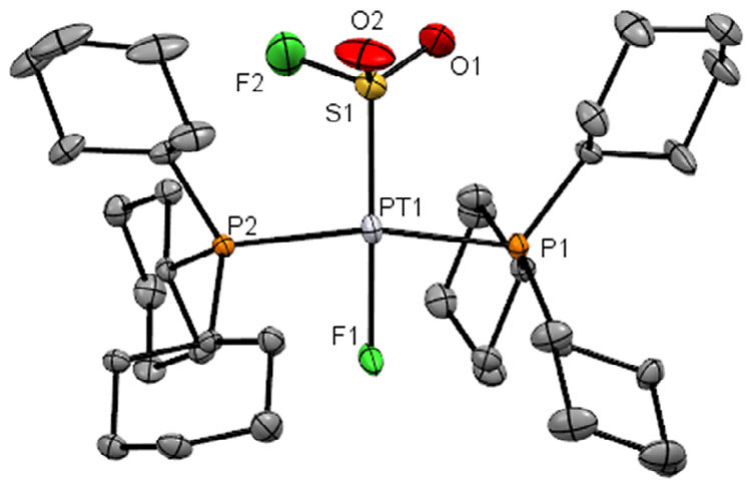
Structure of **4**. Thermal ellipsoids are drawn at 50 % probability level. Carbon‐bound hydrogen atoms were omitted for clarity. Selected distances [Å] and angles [°]: Pt1─S1 2.221(1), Pt1─F1 2.007(2), Pt1─P1 2.353(1), Pt1─P2 2.349(1), S1─F2 1.549(3), S1─O1 1.436(5), and S1─O2 1.496(4); F1─Pt1─S1 179.24(17), F1─Pt1─P1 83.39(3).

Next, the reactivity of **2** toward NFSI was studied. The conversion resulted in the formation of the ionic Pt(II) fluorido compound *trans*‐[Pt(F)(SOF_2_)(PCy_3_)_2_][NFSO_2_Ph] (**5a**) (Scheme 2). By anion exchange with KPF_6_ the fluoroamide anion can be replaced with an PF_6_ anion to form *trans*‐[Pt(F)(SOF_2_)(PCy_3_)_2_][PF_6_] (**5b**). The SOF_2_ complex [Pt(SOF_2_)] has been described in an argon matrix at 25 K.^[^ ^73^ ^]^

**Scheme 2:**
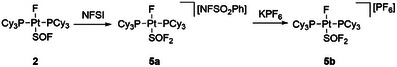
Formation of the complexes **5a** and **5b**.

The ^19^F NMR spectrum of **5a** shows three resonances at chemical shifts of δ = 113, 139, and −265 ppm with an integral ratio of 2:1:1 for the SOF_2_ ligand, the fluoroamide anion [NFSO_2_Ph]^─^,^[^ ^74^ ^]^ and for the metal bound fluorine atom. The latter appears as a triplet of triplets with ^2^ *J*_F,P_ = 29 Hz, ^3^ *J*_F,F_ = 36 Hz, and ^1^ *J*_F,Pt_ = 300 Hz. The fluorine atoms of the SOF_2_ moiety couple with the fluorido ligand and the phosphorus atoms of the phosphine ligands with coupling constants of ^3^ *J*_F,P_ = 5 Hz and ^3^ *J*_F,F_ = 36 Hz (^2^ *J*_F,Pt_ = 750 Hz). Compounds with a SOF_2_ group such as [Hg{C(COF)═SF_2_═O}_2_], R_2_N═S(═O)F_2_ and R_2_S(═O)F_2_ (with R = alkyl and aryl) show signals in the ^19^F NMR for the corresponding fluorine atoms in a range between δ = 46 and 103 ppm.^[^ ^75^, ^76^, ^77^, ^78^, ^79^, ^80^, ^81^ ^]^ The ^31^P{^1^H} NMR spectrum of **5a** reveals one signal with ^195^Pt satellites at δ = 48.4 ppm as a doublet of triplets with ^2^ *J*_P,F_ = 29 Hz, ^3^ *J*_P,F_ = 5 Hz, and ^1^ *J*_P,Pt_ = 1900 Hz. The magnitude of the latter is in good accordance to known phosphorus‐platinum(II) couplings and comparable for the complexes **2** and **4**.^[^ ^32^, ^33^, ^62^, ^71^ ^]^ The NMR spectra of complex **5b** reveal the same data except for the signals of the anion. A solution of the complex *trans*‐[Pt(F)(SOF_2_)(PCy_3_)_2_][PF_6_] (**5b**) in CD_2_Cl_2_ at 298 K produced colorless crystals suitable for X‐ray diffraction (Figure 3). The platinum center of **5b** is in a slightly distorted square‐planar coordination geometry in which both phosphorus atoms of the phosphine ligands are located in a mutually *trans*‐position. The sulfur platinum bond distance of 2.1082(7) Å is shorter when compared to the sulfur platinum bond distance of **4** (2.221(1)) Å, which is probably due to the cationic character of complex **5b**. The bond angles in *trans*‐[Pt(F)(SOF_2_)(PCy_3_)_2_][PF_6_] for the F─S─F unit of 98.2(1)° and the O═S─F entities of 102.4(1)°–104.3(1)° are in accordance to those of SOF_2_ (F─S─F: 92.9(1)°; O═S─F: 105.1(6)°, 105.4(6)°).^[^ ^82^, ^83^ ^]^ For thionyl fluoride the S═O and S─F bond separations of 1.415(2) Å and 1.569(2)–1.575(2) Å, respectively, have been reported. These values correspond closely with the bond lengths observed in compound **5b** (S═O: 1.448(2) Å; S─F: 1.504(2)–1.524(2) Å). An intramolecular C─H···F─Pt interaction in complex **5b** is notable with a short F─C separation of 2.803 Å. Analogous short contacts, with similar F···C distances, have been identified in various other metal fluorido complexes, including Au and Pd.^[^ ^84^, ^85^, ^86^, ^87^ ^]^

**Figure 3:**
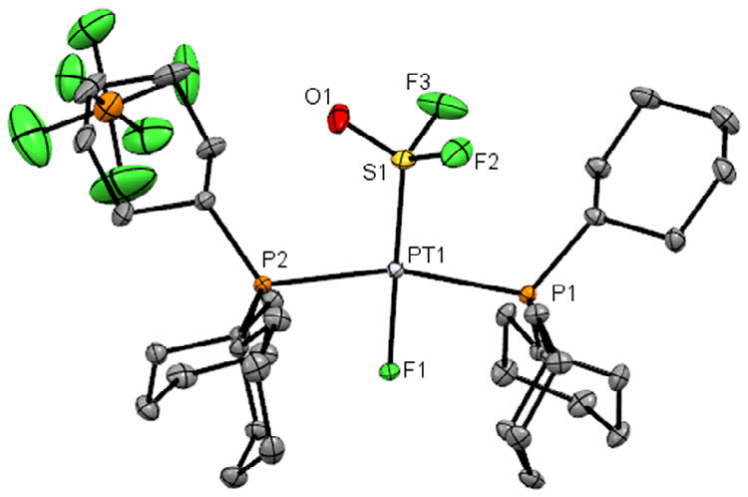
Structure of **5b**. Thermal ellipsoids are drawn at 50 % probability level. Carbon‐bound hydrogen atoms were omitted for clarity. Selected distances [Å] and angles [°]: Pt1─S1 2.1082(7), Pt1─F1 1.952(1), Pt1─P1 2.4001(6), Pt1─P2 2.3973(7), S1─F2 1.543(2), S1─F3 1.516(2), and S1─O1 1.432(2); F1─Pt1─S1 178.04(5), F1─Pt1─P1 82.45(4).

Treatment of *trans*‐[Pt(F)(SOF_2_)(PCy_3_)_2_][NFSO_2_Ph] (**5a**) with TMAF (Me_4_NF) in CD_2_Cl_2_ led after 1 h at 253 K to the formation of a platinum(II) complex for which we suggest the structure *trans*‐[Pt(F)(SOF_3_)(PCy_3_)_2_] (**6**) bearing a sulfur bound SOF_3_ ligand (Scheme 3).^[^ ^88^ ^]^ Though, **6** starts to decompose at 283 K to form the difluorido platinum complex *trans*‐[Pt(F)_2_(PCy_3_)_2_] (**7**) (for X‐ray studies of **7**, see Supporting Information) and SOF_2_.

**Scheme 3:**
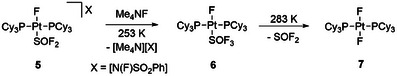
Formation of the complexes **6** and **7**.

The ^19^F NMR spectra of **6** at 253 K shows two signals for the SOF_3_ ligand and one for the fluorido ligand, which integrate in a ratio of 2:1:1. The sulfur bound fluorine atoms give a signal at *δ* = 163 ppm for the two fluorine atoms in the apical position and one signal at δ = 95 ppm for the fluorine atom in the equatorial plane of a trigonal‐bipyramidal arrangement at the sulfur center. The former signal appears as a doublet of doublet of multiplets (^2^ *J*_F,F_ = 125 Hz, ^3^ *J*_F,F_ = 44 Hz, and ^1^ *J*_F,Pt_ = 860 Hz), whereas the latter is a triplet of doublet of multiplets (^2^ *J*_F,F_ = 125 Hz, ^3^ *J*_F,F_ = 23 Hz, and ^1^ *J*_F,Pt_ = 440 Hz). The magnitude of coupling constants is in good accordance to known fluorine‐fluorine *cis* couplings at the sulfur atom.^[^ ^89^, ^90^ ^]^ The mutual coupling of the fluorine atoms was additionally confirmed by a ^19^F, ^9^F‐COSY NMR spectrum. For the metal fluorido ligand a signal at δ = −353 ppm was observed at 253 K. The ^31^P{^1^H} NMR spectrum (242.9 MHz) of **6** at 253 K, displays a system of higher‐order with signals at *δ* = 29.2 ppm (^2^ *J*_Pa,Pb_ = 325 Hz, ^3^ *J*_Pa,F_ = 23.9 Hz) and δ = 26.1 ppm (^2^ *J*_Pb,Pa_ = 325 Hz, ^3^ *J*_Pb,F_ = 23.1 Hz), for two magnetically nonequivalent phosphorus nuclei (for simulation see Supporting Information). The phosphorus–phosphorus coupling constant of ^2^ *J*_P,P_ ═ 325 Hz is consistent with phosphine moieties occupying a mutually *trans* configuration.^[^ ^91^, ^92^ ^]^ The nonequivalence of the two phosphorus nuclei is likely attributed to a hindered rotation about the Pt─S bond. Variable temperature ^31^P{^1^H} and ^19^F NMR spectra reveal the appearance of a second set of signals and the spectra at 183 K show resonances with comparable chemical shifts and similar coupling constants. This can be due to two distinct arrangements of the SOF_3_ ligand, or the presence of a derivative which is possibly characterized by any donor interaction to the metal bound fluoride.^[^ ^62^, ^63^, ^68^, ^86^, ^93^, ^94^, ^95^, ^96^, ^97^ ^]^

DFT calculations of **6** support the trigonal‐bipyramidal configuration at the metal bound sulfur atom. An isomer (**6′**), featuring oxygen‐coordination of the OSF_3_ ligand to Pt, is 13.4 kJ mol^─1^ higher in energy relative to **6** (Figure 4). Furthermore, the isomer **6′′** (Figure S27, Supporting Information), which is characterized by a sulfur‐bonding to Pt and an axial arrangement of a fluorine and oxygen atom at the ligand, is significantly destabilized and exhibits an energy that is 88.3 kJ mol^─1^ higher than **6**. Note also that the observed coupling constant of ^2^ *J*_F,F_ ═ 125 Hz in the ^19^F NMR spectrum of **6** is inconsistent with a structure featuring an O‐bound Pt─OSF_3_ ligand. Literature precedents for R─OSF_3_ species typically exhibit significantly smaller ^2^ *J*_F,F_ values.^[^ ^98^, ^99^, ^100^, ^101^, ^102^, ^103^ ^]^

**Figure 4:**
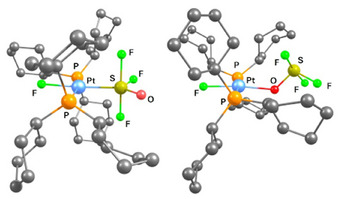
DFT optimized structures of the complex **6** (left) and **6′** (right); B3LYP‐D3(BJ)/cc‐pvdz (RECP with corresponding cc‐pvdz basis set for Pt). All hydrogen atoms have been omitted for clarity.

In conclusion, this study reports on the development of unique routes for the stabilization of sulfur oxofluorides at transition metal centers by oxygenation as well as consecutive electrophilic or nucleophilic fluorination steps. The synthetic strategies pave the way for the preparation of a wider range of metal complexes with high‐valent sulfur fluoride ligands. The identification of platinum complexes bearing SO_2_F, SOF_2_, and remarkably SOF_3_ ligands demonstrate the feasibility for stabilizing these highly reactive species within the coordination sphere of a metal. The identification of the SOF_3_ complex is exceptional, as it represents a sole example of a high‐valent fluorinated sulfur atom coordinated to a metal center. The complexes hold promise as possible transfer reagents for sulfur fluoride entities.

## Supporting Information

The authors have cited additional references within the Supporting Information.^[^ ^24^, ^32^, ^104^, ^105^, ^106^, ^107^, ^108^, ^109^, ^110^, ^111^, ^112^, ^113^ ^]^

## Conflict of Interests

The authors declare no conflict of interest.

## Supporting information



Supporting Information

## Data Availability

The data that support the findings of this study are available in the Supporting Information of this article.
