# Supplementary material for: Routes to Pt Derivatives of High‐Valent Sulfur Oxofluorides S(═O)2F, S(═O)F2, and S(═O)F3 by Fluorination and Oxygenation
Source: Angew Chem Int Ed Engl. 2025 Apr 7;64(23):e202503153. doi: 10.1002/anie.202503153 (PMC12124436; doi:10.1002/anie.202503153)
Supplement: Supplementary file 1 — Supporting Information [file ANIE-64-e202503153-s001.pdf]

Routes to Pt Derivatives of High-valent Sulfur Oxofluorides $S(=O)_2F$, $S(=O)F_2$ and $S(=O)F_3$ by Fluorination and Oxygenation

Ruben Jaeger, Ouchan He, Stefan Sander, Dilcan Dirican, Mike Ahrens and Thomas Braun*

Humboldt-Universität zu Berlin, Department of Chemistry, Brook-Taylor-Straße 2, 12489 Berlin (Germany)

Table of Contents

Table of Contents ... 1

1. General Information ... 2
2. Generation of *trans*-$[Pt(F)(SO_2F)(PCy_3)_2]$ (4) ... 3
3. Alternative syntheses of *trans*-$[Pt(F)(SO_2F)(PCy_3)_2]$ (4) ... 3
4. Generation of *trans*-$[Pt(F)(SOF_2)(PCy_3)_2][NFSO_2Ph]$ (5a) ... 4
5. Generation of *trans*-$[Pt(F)(SOF_2)(PCy_3)_2][PF_6]$ (5b) ... 4
6. Generation of *trans*-$[Pt(F)(SOF_3)(PCy_3)_2]$ (6) ... 5
7. Generation of *trans*-$[Pt(F)_2(PCy_3)_2]$ (7) ... 6
8. NMR Spectra ... 7
9. Variable-temperature studies of *trans*-$[Pt(F)(SOF_3)(PCy_3)_2]$ (6) ... 16
10. Structures and Crystallographic Data ... 19
11. Computational Details ... 23
12. References ... 30

1. General Information

The synthetic work was carried out with a Schlenk line or in a glove box in an atmosphere of argon. All reactions with SO_2F_2 and were performed on a stainless-steel vacuum line. The PFA tubes used are those with an outer diameter of 3.8 mm. All solvents were purified and dried by conventional methods and distilled under an atmosphere of argon before use. $[Pt(PCy_3)_2]$ (**1**), $[Pt(SO_2)(PCy_3)_2]$, and *trans*-$[Pt(F)(SOF)(PCy_3)_2]$ (**2**) were prepared according to literature procedure.[1] All reagents were obtained from commercial sources and were used without further purification. The NMR spectra were acquired at a Bruker DPX 300, Bruker Avance II 300, Bruker Avance III 300 or a Bruker Avance 600 NMR spectrometer. The ^1H chemical shifts were referenced to residual benzene-d$_5$ at δ = 7.160 ppm, dichloromethane-d$_1$ at δ = 5.32 ppm or tetrahydrofuran-d$_7$ at δ = 3.580 ppm. The ^{19}F NMR spectra were referenced to external $CFCl_3$ at δ = 0.0 ppm and the $^{31}P\{^1H\}$ NMR spectra to external 85% H_3PO_4 at δ = 0.0 ppm. ATR-IR spectra were recorded inside a glovebox on a Bruker ALPHA II spectrometer equipped with an ATR-module (diamond).

Structure determination of complexes **2**, **4**, **5b**, **7**: Colorless crystals of **2**, **4** and **5b** have been obtained by gas-phase diffusion of *n*-pentane into the reaction solutions at room temperature. Colorless crystals of **7** have been obtained by slow evaporation at 233 K out of the reaction solution. The diffraction data were collected at a Bruker D8 Venture diffractometer at 100 K using Mo-Kα (λ = 0.71073 Å) radiation. Multi-scan absorption corrections implemented SADABS were applied to the data.[2] The structures were solved by intrinsic phasing method (SHELXT 2014/5)[3] and refined by full-matrix least-squares methods on F2 (SHELXL 2016/4 or SHELXL-2018/3).[4] All hydrogen atoms were placed at calculated positions and refined using a riding model. Deposition Numbers CCDC-2432051 (for **2**), CCDC-2432045 (for **4**), CCDC-2432048 (for **5b**) and CCDC-2432052 (for **7**) contain the supplementary crystallographic data for this paper. These data are provided free of charge by the joint Cambridge Crystallographic Data Centre and Fachinformationszentrum Karlsruhe Access Structures service.

Computational details: DFT-calculations for the compounds **6**, **6'** and **6''** were performed using the Gaussian 16 (Revision A.03) program package[5] and the B3LYP functional; cc-pvtz basis sets were employed for all atoms except for rhodium, which was described on using a RECP with the associated cc-pvdz basis set.[6] The calculated electron density was further analyzed using the program Multiwfn.[7] Dispersion effects were taken into account using the Grimme D3 dispersion correction with Becke-Johnson damping.[8] Frequency calculations were run for all stationary points to identify them as minima (no negative eigenvalues). Energies were corrected for zero-point energy.

2. Generation of *trans*-[Pt(F)(SO_2F)(PCy_3)$_2$] (4)

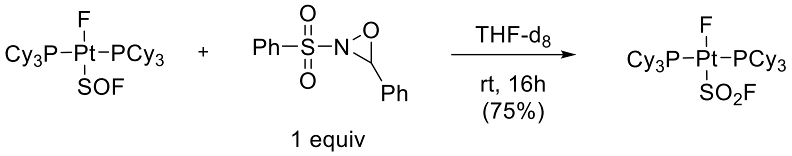

2 **4**

A PFA tube placed inside a Young NMR tube was loaded with Davis reagent (3-Phenyl-2-(phenylsulfonyl)-1,2-oxaziridine) (16 mg, 0.6 mmol) and *trans*-[Pt(F)(SOF)(PCy_3)$_2$] (**2**) (50 mg, 0.06 mmol). After adding deuterated tetrahydrofuran, the reaction mixture was monitored by NMR spectroscopy. After 16h the $^{31}P\{^1H\}$ and ^{19}F NMR spectroscopic data revealed a complete conversion to **4**. The solvent was removed in vacuum and the off-white solid was washed with small portions of pentane. After drying in vacuum, complex **4** was obtained as an off-white powder (36.6 mg, 0.043 mmol, 75%).

^{1}H NMR (300 MHz, THF-d_8) δ = 2.13-0.98 (m) ppm. **^{19}F NMR** (282.4 MHz, THF-d_8): δ = 126 (td + sat, $^3J_{F,P}$ = 4 Hz, $^3J_{F,F}$ = 43 Hz, $^2J_{F,Pt}$ = 980 Hz, 1F, SO_2F); -310 (td, $^2J_{F,P}$ = 22 Hz, $^3J_{F,F}$ = 43 Hz, 1F, PtF) ppm. **^{31}P{^{1}H} NMR** (121.5 MHz, THF-d_8): δ = 33.8 (dd + sat, $^2J_{P,F}$ = 22 Hz, $^3J_{P,F}$ = 4 Hz, $^1J_{P,Pt}$ = 2500 Hz, PtP) ppm. **Elemental analysis** (%) calc. for $C_{36}H_{66}F_2O_2P_2PtS$: calc.: C 50.40, H 7.75; S 3.74; found: C 50.80, H 7.85, S 3.95. **IR** (ATR, diamond): $\tilde{\nu}$ = 493 (m, Pt–F) cm^{-1}.[1c,9]

3. Alternative syntheses of *trans*-[Pt(F)(SO_2F)(PCy_3)$_2$] (4)

(a) A PFA tube placed inside a Young NMR tube was loaded with NFSI (*N*-Fluorobenzenesulfonimide) (19 mg, 0.06 mmol) and [Pt(SO_2)(PCy_3)$_2$] (25 mg, 0.03 mmol). The NFSI contained considerable amounts of HF. After adding deuterated tetrahydrofuran, the reaction mixture was monitored by NMR spectroscopy. After 16h the $^{31}P\{^1H\}$ and ^{19}F NMR spectroscopic data revealed a conversion to **4**. The solvent was removed in vacuum and the off-white solid was washed with small portions of pentane. After drying in vacuum, complex **4** was obtained as an off-white powder (9.1 mg, 0.01 mmol, 30%).

(b) Freshly dried and degassed deuterated tetrahydrofuran was condensed at 77 K into a PFA tube loaded with [Pt(PCy_3)$_2$] (**1**) (50 mg, 0.08 mmol). The solution was degassed in vacuo and 1 equiv. of SO_2F_2 was condensed into the PFA tube at 77 K. The PFA tube was sealed and the reaction mixture was warmed up to room temperature. The reaction mixture was monitored by NMR spectroscopy. After 16h the $^{31}P\{^1H\}$ and ^{19}F NMR spectroscopic data revealed a conversion to **4**. The PFA tube was cut open, the solvent was removed in vacuum and the off-white solid was washed with small portions of pentane. After drying in vacuum, complex **4** was obtained as an off-white powder (19.7 mg, 0.023 mmol, 35%).

4. Generation of *trans*-[Pt(F)(SOF_2)(PCy_3)$_2$][$NFSO_2Ph$] (5a)

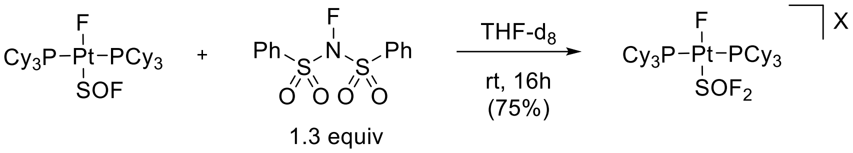

2 **5a**

X = $NFSO_2Ph$

A PFA tube placed inside a Young NMR tube was loaded with NFSI (*N*-Fluorobenzenesulfonimide) (13 mg, 0.04 mmol) and *trans*-[Pt(F)(SOF)(PCy_3)$_2$] (**2**) (25 mg, 0.03 mmol). After adding deuterated tetrahydrofuran, the reaction mixture was monitored by NMR spectroscopy. After 16h the $^{31}P\{^1H\}$ and ^{19}F NMR spectroscopic data revealed a complete conversion to **5a**. The reaction mixture was filtered, the solvent was removed in vacuum and the off-white solid was washed with small portions of pentane. After drying in vacuum, complex **5a** was obtained as an off-white powder with (18.6 mg, 0.018 mmol, 75%).

^{1}H NMR (300 MHz, THF-d$_8$) δ = 2.88-1.25 (m) ppm. **^{19}F NMR** (282.4 MHz, THF-d$_8$): δ = 113 (td + sat, $^3J_{F,P}$ = 5 Hz, $^3J_{F,F}$ = 36 Hz, $^2J_{F,Pt}$ = 750 Hz, 2F, SOF_2); -139 (s, 1F, NF); -265 (tt + sat, $^2J_{F,P}$ = 29 Hz, $^3J_{F,F}$ = 36 Hz, $^1J_{F,Pt}$ = 300 Hz, 1F, PtF) ppm. **^{31}P{^{1}H} NMR** (121.5 MHz, THF-d$_8$): δ = 48.4 (dt + sat, $^2J_{P,F}$ = 29 Hz, $^3J_{P,F}$ = 5 Hz, $^1J_{P,Pt}$ = 1900 Hz, PtP) ppm. **IR** (ATR, diamond): ṽ = 494 (m, Pt–F) cm^{-1}.

5. Generation of *trans*-[Pt(F)(SOF_2)(PCy_3)$_2$][PF_6] (5b)

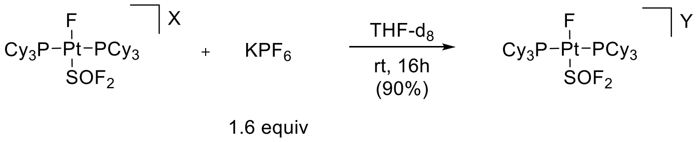

5a **5b**

X = $NFSO_2Ph$
Y = PF_6

A solution of *trans*-[Pt(F)(SOF_2)(PCy_3)$_2$][$NFSO_2Ph$] (**5a**) (30 mg, 0.03 mmol) in deuterated tetrahydrofuran in a PFA tube placed inside a Young NMR tube was loaded with KPF_6 (9.2 mg, 0.05 mmol). After 16h the reaction mixture was filtered, the solvent was removed in vacuum and the off-

white solid was washed with small portions of pentane. After drying in vacuum, the complex *trans*-$[Pt(F)(SOF_2)(PCy_3)_2][PF_6]$ (**5b**) was obtained as an off-white powder (27.2 mg, 0.027 mmol, 90%).

^{1}H NMR (300 MHz, THF-d_8) δ = 2.88-1.25 (m) ppm. **^{19}F NMR** (282.4 MHz, THF-d_8): δ = 113 (td + sat, $^3J_{F,P}$ = 5 Hz, $^3J_{F,F}$ = 36 Hz, $^2J_{F,Pt}$ = 750 Hz, 2F, SOF_2); -74 (d, $^1J_{F,P}$ = 710 Hz, 6F, PF_6); -265 (tt + sat, $^2J_{F,P}$ = 29 Hz, $^3J_{F,F}$ = 43 Hz, $^1J_{F,Pt}$ = 300 Hz, 1F, PtF) ppm. **^{31}P{^{1}H} NMR** (121.5 MHz, THF-d_8): δ = 48.4 (dt + sat, $^2J_{P,F}$ = 29 Hz, $^3J_{P,F}$ = 5 Hz, $^1J_{P,Pt}$ = 1900 Hz, PtP); -144 ppm (spt, $^1J_{P,F}$ = 710 Hz, 1P, PF_6) ppm.

6. Generation of *trans*-$[Pt(F)(SOF_3)(PCy_3)_2]$ (6)

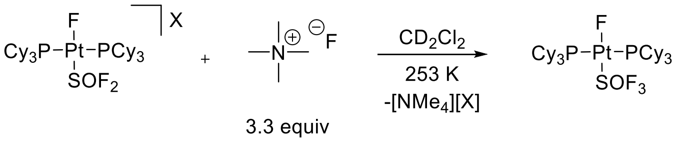

5a **6**

X = $NFSO_2Ph$

A solution of *trans*-$[Pt(F)(SOF_2)(PCy_3)_2][NFSO_2Ph]$ (30 mg, 0.03 mmol) in CD_2Cl_2 placed in a PFA tube inside a Young NMR tube was cooled to 233 K and loaded with Me_4NF (10 mg, 0.10 mmol). Compound **6** was characterized by low-temperature NMR spectroscopy.

^{1}H NMR (300 MHz, CD_2Cl_2, 253 K) δ = 2.28-1.25 (m) ppm. **^{19}F NMR** (282.4 MHz, CD_2Cl_2, 253 K): δ = 163 (dd + sat, $^2J_{F,F}$ = 125 Hz, $^3J_{F,F}$ = 44 Hz, $^2J_{F,Pt}$ = 860 Hz, 2F(ax.), SOF_3); 95 (td + sat, $^2J_{F,F}$ = 125 Hz, $^3J_{F,F}$ = 23 Hz, $^2J_{F,Pt}$ = 440 Hz, 1F(equ.), SOF_3); -353 (s, br + sat; $^1J_{F,Pt}$ = 270 Hz, 1F, PtF) ppm. **^{31}P{^{1}H} NMR** (121.5 MHz, CD_2Cl_2, 253 K): δ = 29.2 ($^2J_{Pa,Pb}$ = 325 Hz, $^3J_{Pa,F}$ = 23.9 Hz, $^3J_{F(ax),Pa}$ = 5.5 Hz, $^3J_{F(eq),Pa}$ = 8.8 Hz, $^1J_{Pa,Pt}$ = 2460 Hz, 1P_a); 26.1 ($^2J_{Pb,Pa}$ = 325 Hz, $^3J_{Pb,F}$ = 23.1 Hz, $^3J_{F(ax),Pb}$ = 4.5 Hz, $^3J_{F(eq),Pb}$ = 5.5 Hz, $^1J_{Pb,Pt}$ = 2460 Hz, 1P_b) ppm. The chemical shifts and the coupling constants of the ^{31}P{^{1}H} NMR spectrum of **6** were obtained by simulation with gNMR.[10]

7. Generation of *trans*-[Pt(F)$_2$(PCy$_3$)$_2$] (7)

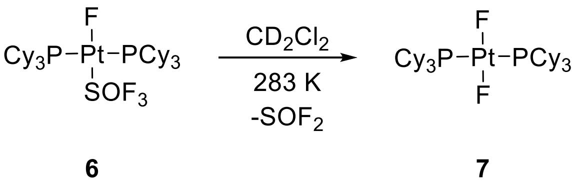

A reaction solution in CD_2Cl_2 (0.5 ml) of *trans*-[Pt(F)(SOF$_3$)(PCy$_3$)$_2$] (**6**) (0.028 mmol) in a PFA tube placed inside a Young NMR tube was warmed to 283 K. The reaction mixture was monitored NMR spectroscopy at 283 K to reveal the generation of **7**.

^{1}H NMR (300 MHz, CD_2Cl_2, 283 K) δ = 2.28-1.25 (m) ppm. **^{19}F NMR** (282.4 MHz, CD_2Cl_2, 283 K): δ = -453 (t + sat, $^{2}J_{F,P}$ = 11 Hz; $^{1}J_{F,Pt}$ = 973 Hz, 2F, PtF) ppm. **^{31}P{^{1}H} NMR** (121.5 MHz, CD_2Cl_2, 283 K): δ = 24.9 (t + sat, $^{2}J_{P,F}$ = 11 Hz, $^{1}J_{P,Pt}$ = 2820 Hz, PtP) ppm.

8. NMR Spectra

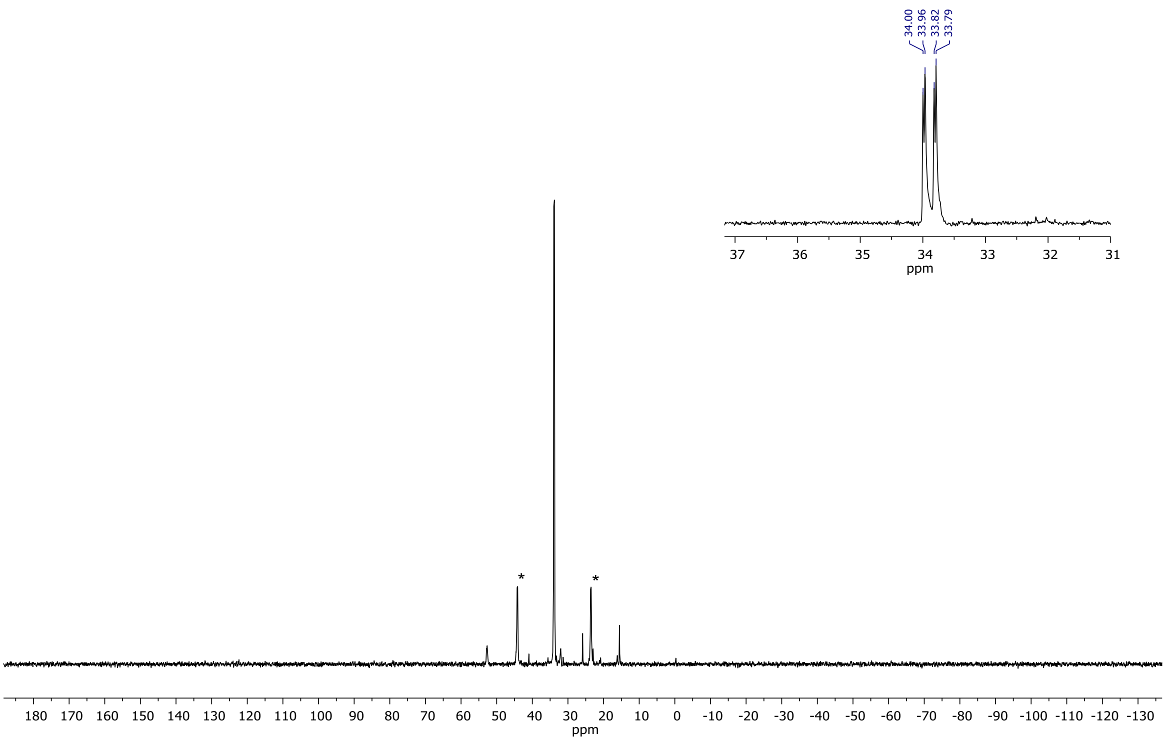

Figure S1. $^{31}P\{^{1}H\}$ NMR spectrum of *trans*-[Pt(F)(SO_2F)(PCy_3)$_2$] (**4**), * ^{195}Pt satellites (THF-d_8, 121.5 MHz); minor impurities consist i.a. of phosphine oxide.

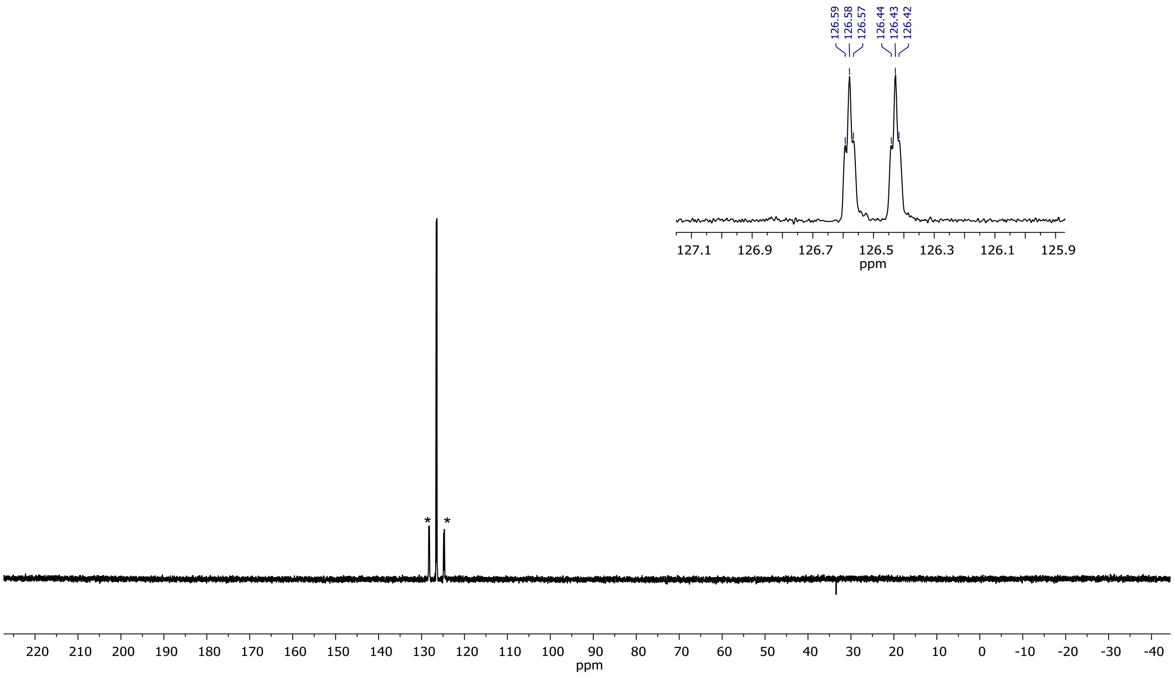

Figure S2. ^{19}F NMR spectrum of *trans*-[Pt(F)(SO_2F)(PCy_3)$_2$] (**4**), * ^{195}Pt satellites (THF-d_8, 282 MHz).

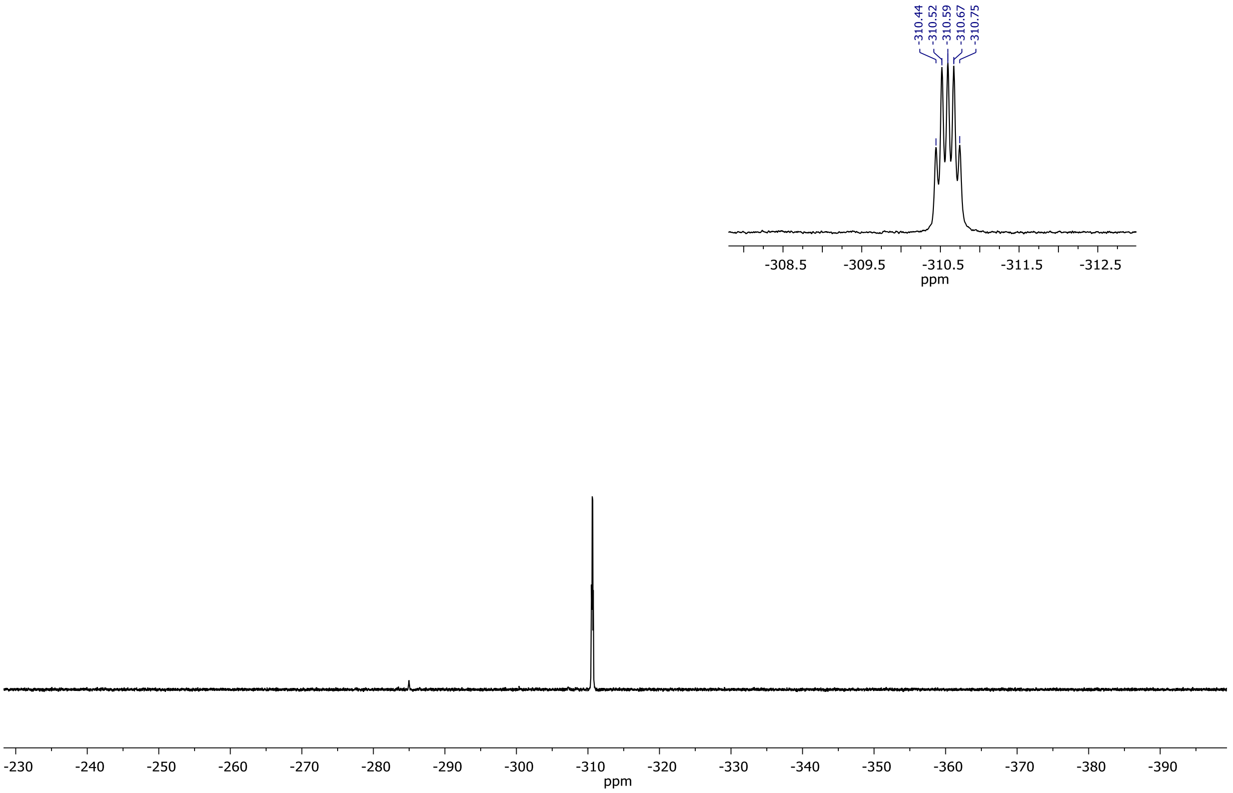

Figure S3. ^{19}F NMR spectrum of *trans*-[Pt(F)(SO_2F)(PCy_3)$_2$] (**4**) (THF-d_8, 282 MHz). Simulation reveal that the Pt satellites can be covered by the main signal.[10]

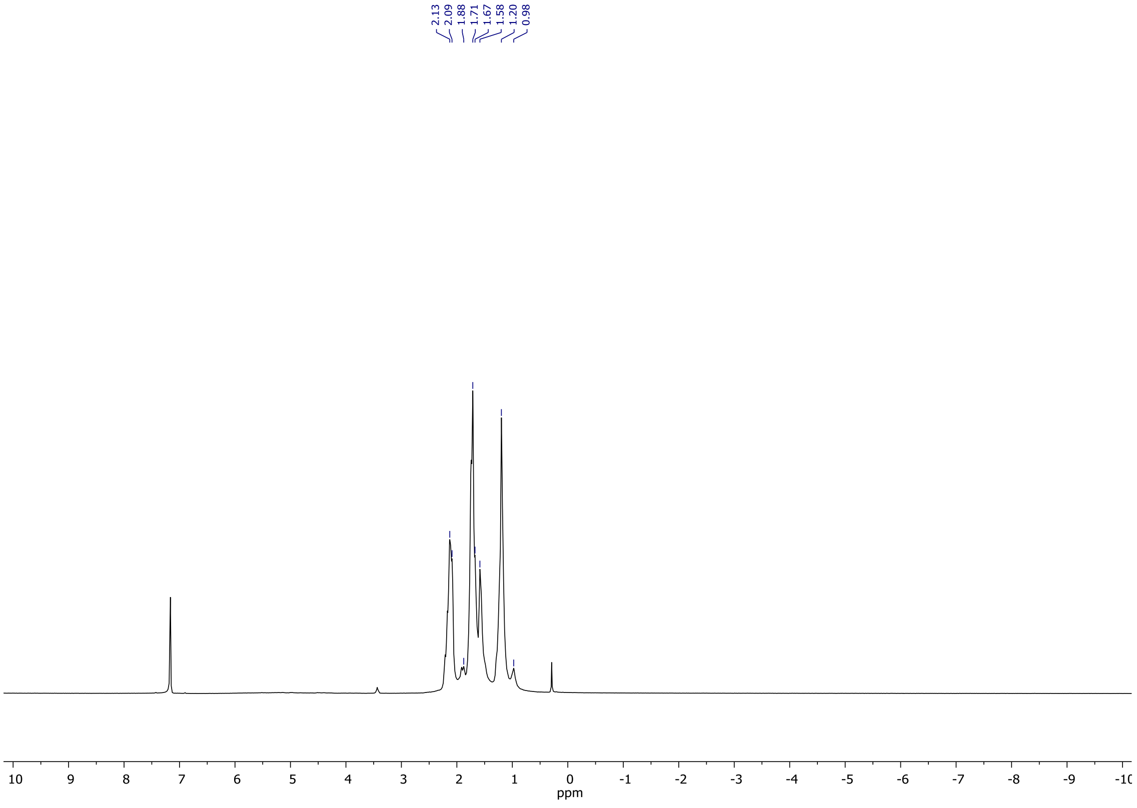

Figure S4. ^{1}H NMR spectrum of *trans*-[Pt(F)(SO_2F)(PCy_3)$_2$] (**4**) (C_6D_6, 300 MHz).

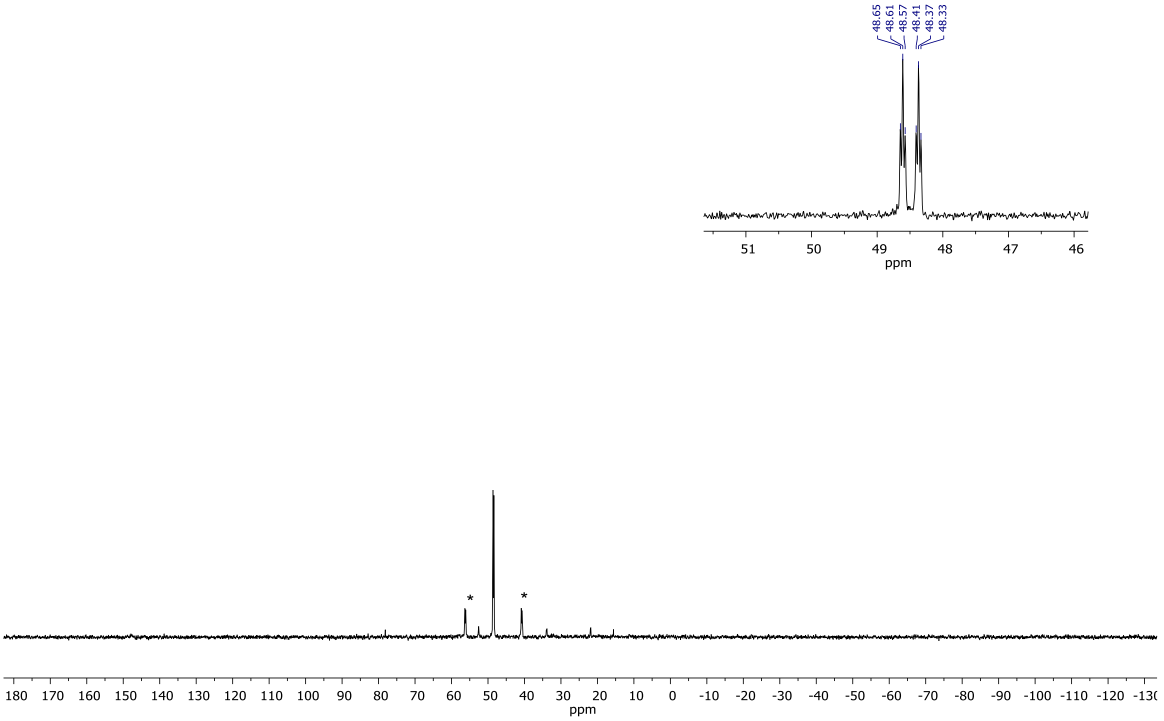

Figure S5. $^{31}P\{^{1}H\}$ NMR spectrum of *trans*-$[Pt(F)(SOF_2)(PCy_3)_2][NFSO_2Ph]$ (**5a**), * ^{195}Pt satellites (THF-d_8, 121.5 MHz).

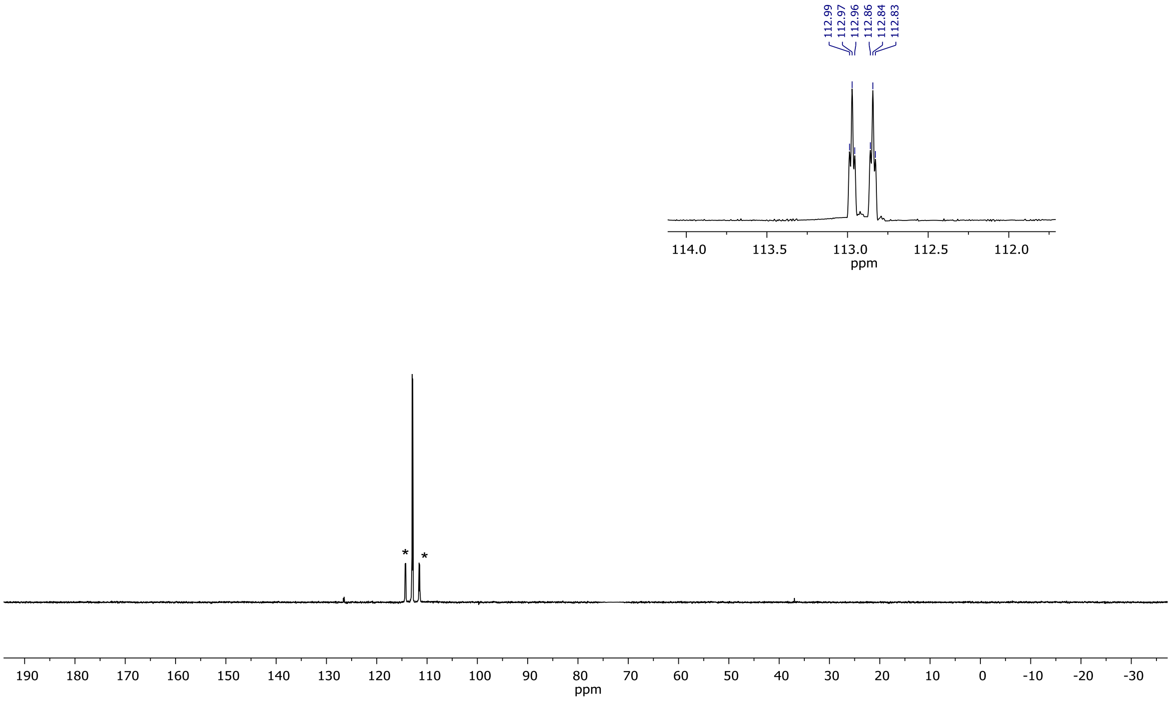

Figure S6. ^{19}F NMR spectrum of *trans*-$[Pt(F)(SOF_2)(PCy_3)_2][NFSO_2Ph]$ (**5a**), * ^{195}Pt satellites (THF-d_8, 282 MHz).

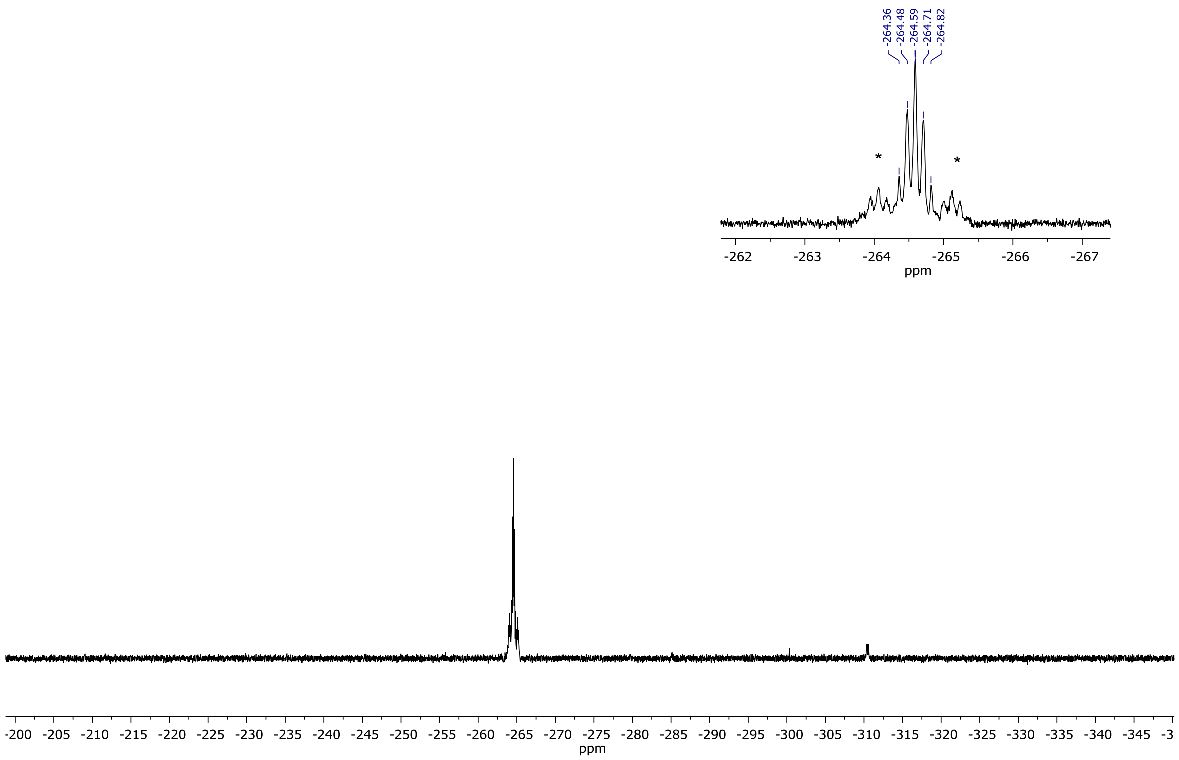

Figure S7. ^{19}F NMR spectrum of *trans*-[Pt(F)(SOF_2)(PCy_3)$_2$][$NFSO_2Ph$] (**5a**), * ^{195}Pt satellites (THF-d_8, 282 MHz).

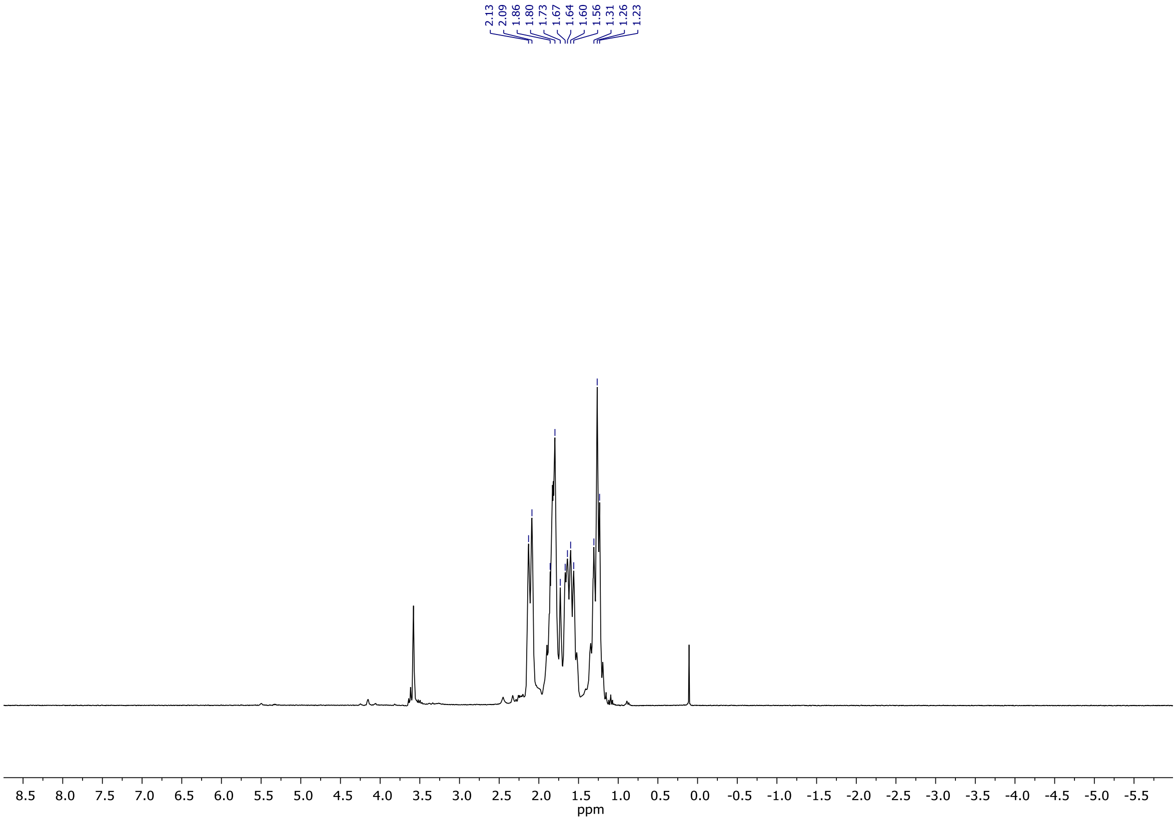

Figure S8. ^{1}H NMR spectrum of *trans*-[Pt(F)(SOF_2)(PCy_3)$_2$][$NFSO_2Ph$] (**5a**) (THF-d_8, 300 MHz).

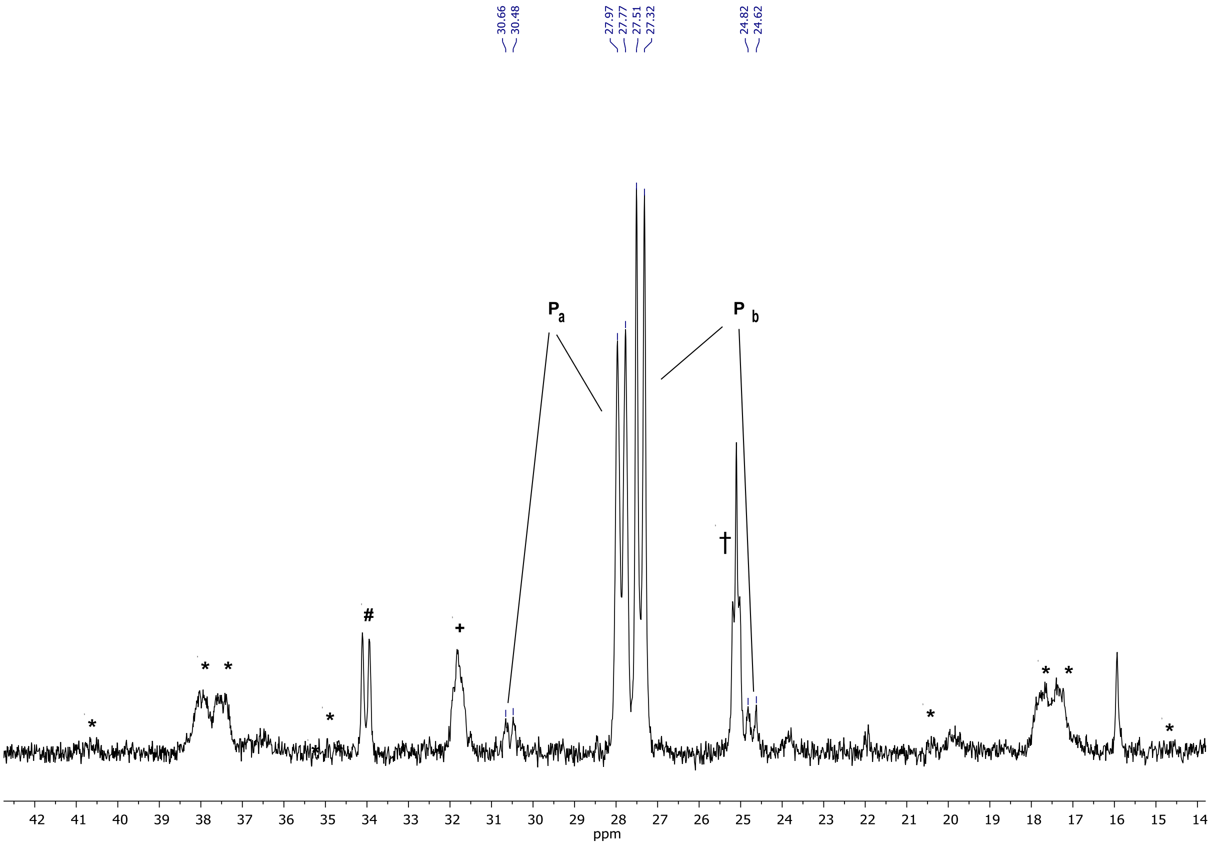

Figure S9. $^{31}P\{^{1}H\}$ NMR spectrum of *trans*-$[Pt(F)(SOF_3)(PCy_3)_2]$ (**6**) at 253 K, * ^{195}Pt satellites, + *trans*-$[Pt(F)(SOF)(PCy_3)_2]$ (**2**), # *trans*-$[Pt(F)(SO_2F)(PCy_3)_2]$ (**4**), † *trans*-$[Pt(F)_2(PCy_3)_2]$ (**7**) (CD_2Cl_2, 121.5 MHz).

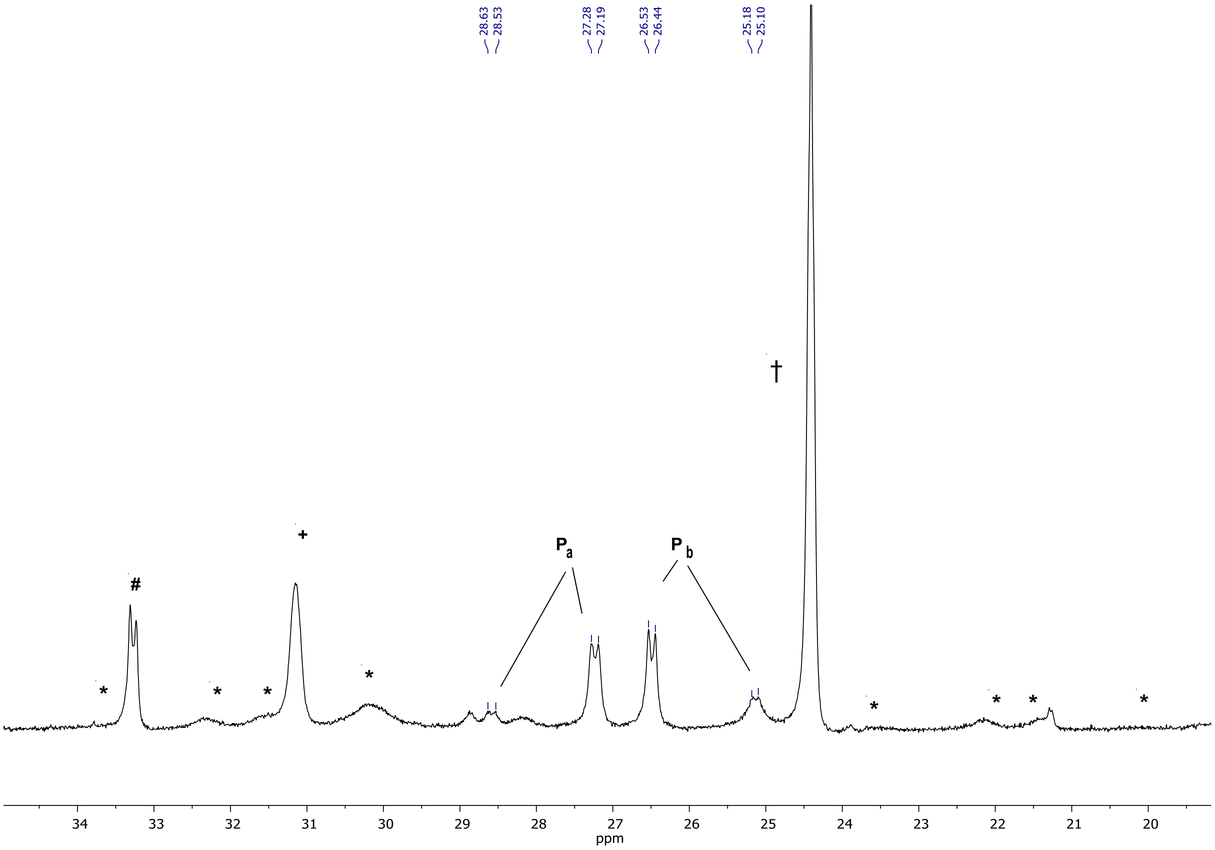

Figure S10. $^{31}P\{^{1}H\}$ NMR spectrum of *trans*-[Pt(F)(SOF$_3$)(PCy$_3$)$_2$] (**6**) at 263 K, * ^{195}Pt satellites, + *trans*-[Pt(F)(SOF)(PCy$_3$)$_2$] (**2**), # *trans*-[Pt(F)(SO$_2$F)(PCy$_3$)$_2$] (**4**), † *trans*-[Pt(F)$_2$(PCy$_3$)$_2$] (**7**) (CD$_2$Cl$_2$, 242.9 MHz).

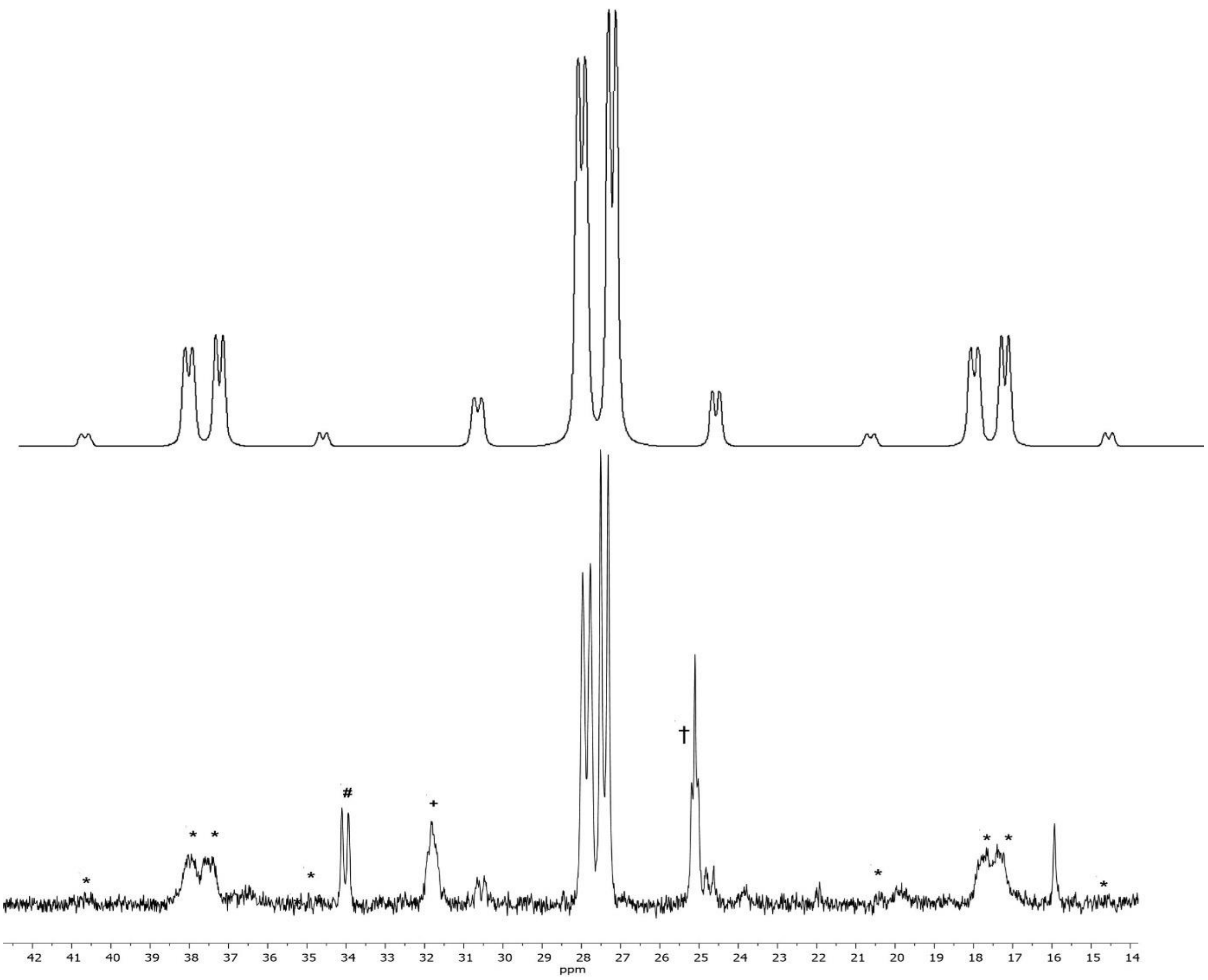

Figure S11. $^{31}P\{^{1}H\}$ NMR spectrum of *trans*-[Pt(F)(SOF$_3$)(PCy$_3$)$_2$] (**6**); top: simulated spectrum[10]; bottom: recorded at 253 K, * ^{195}Pt satellites, + *trans*-[Pt(F)(SOF)(PCy$_3$)$_2$] (**2**), # *trans*-[Pt(F)(SO$_2$F)(PCy$_3$)$_2$] (**4**), † *trans*-[Pt(F)$_2$(PCy$_3$)$_2$] (**7**) CD$_2$Cl$_2$, 121.5 MHz).

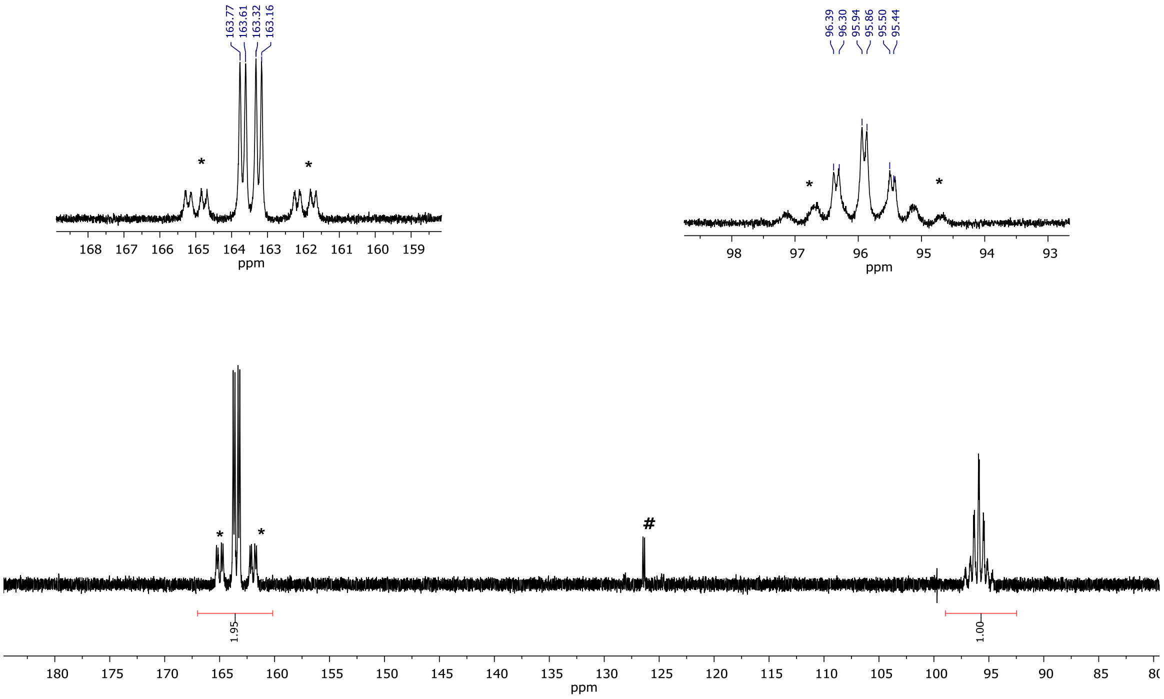

Figure S12. ^{19}F NMR spectrum of *trans*-[Pt(F)(SOF_3)(PCy_3)$_2$] (**6**) at 253 K, * ^{195}Pt satellites, # *trans*-[Pt(F)(SO_2F)(PCy_3)$_2$] (**4**) (CD_2Cl_2, 282 MHz).

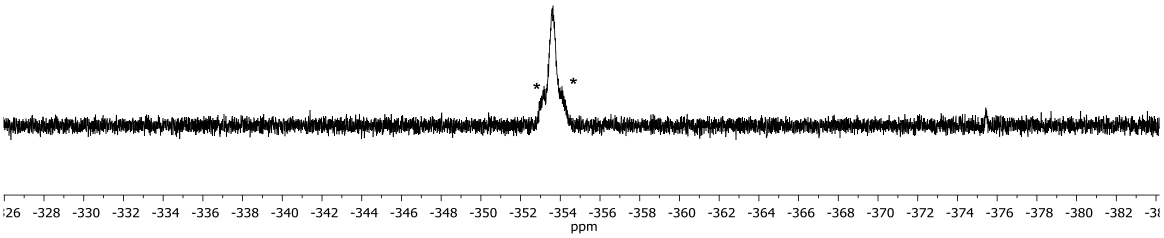

Figure S13. ^{19}F NMR spectrum of *trans*-[Pt(F)(SOF_3)(PCy_3)$_2$] (**6**) at 253 K, * ^{195}Pt satellites (CD_2Cl_2, 282 MHz).

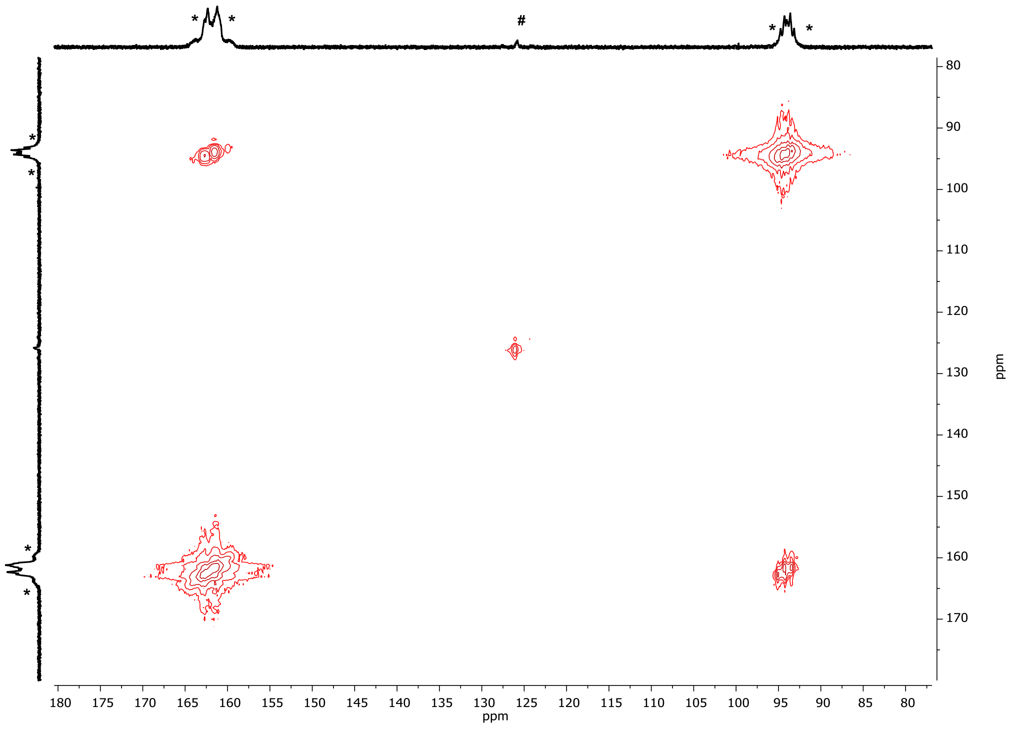

Figure S14. $^{19}F/^{19}F$ COSY NMR spectrum of *trans*-[Pt(F)(SOF$_3$)(PCy$_3$)$_2$] (**6**) at 203 K, * ^{195}Pt satellites, # *trans*-[Pt(F)(SO$_2$F)(PCy$_3$)$_2$] (**4**) (CD_2Cl_2, 282 MHz).

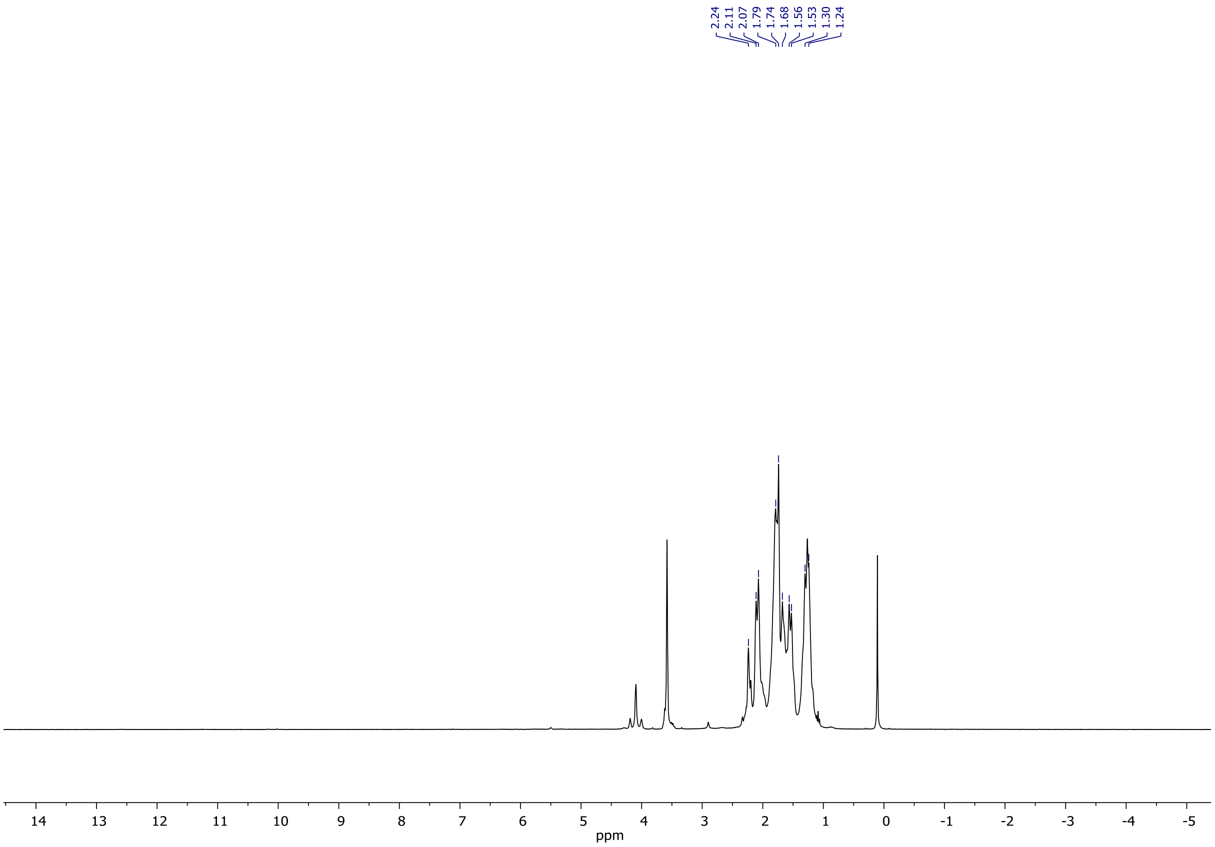

Figure S15. ^{1}H NMR spectrum of *trans*-[Pt(F)(SOF$_3$)(PCy$_3$)$_2$] (**6**) at 253 K (THF-d$_8$, 300 MHz).

9. Variable-temperature studies of *trans*-[Pt(F)(SOF$_3$)(PCy$_3$)$_2$] (6)

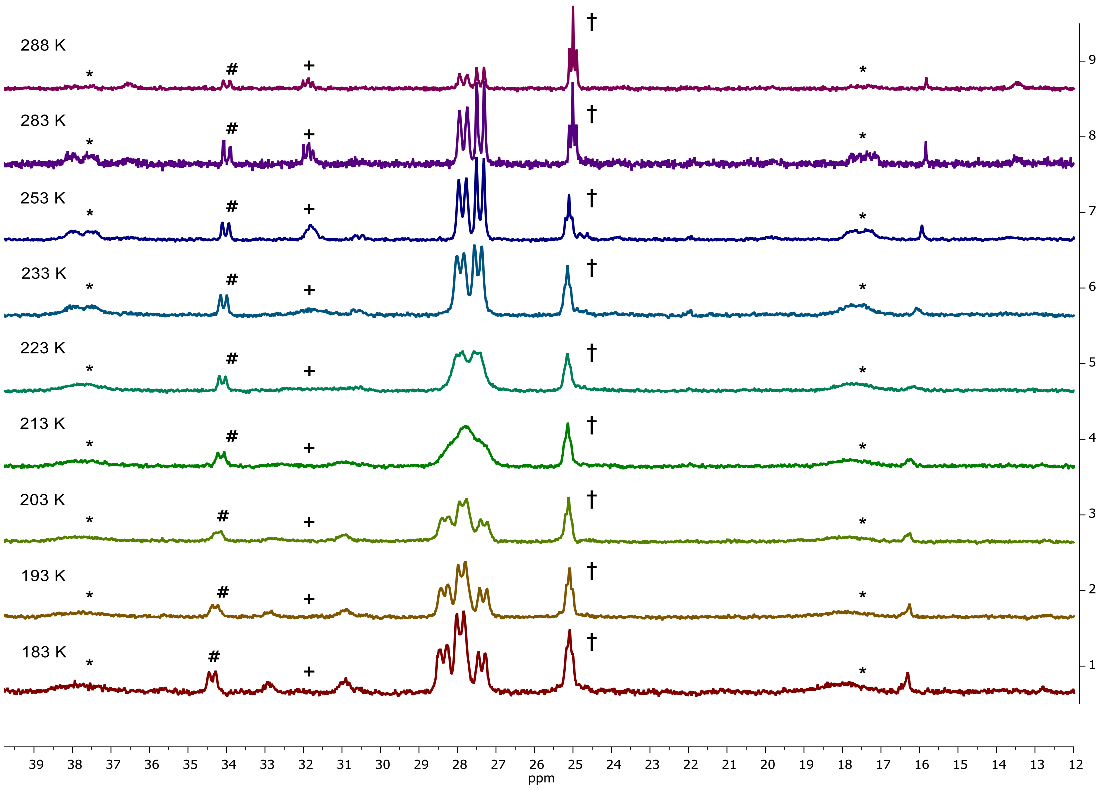

Figure S16. Variable-temperature $^{31}P\{^{1}H\}$ NMR spectra of *trans*-[Pt(F)(SOF$_3$)(PCy$_3$)$_2$] (**6**), * ^{195}Pt satellites, + *trans*-[Pt(F)(SOF)(PCy$_3$)$_2$] (**2**), # *trans*-[Pt(F)(SO$_2$F)(PCy$_3$)$_2$] (**4**), † *trans*-[Pt(F)$_2$(PCy$_3$)$_2$] (**7**), (CD$_2$Cl$_2$, 121.5 MHz).

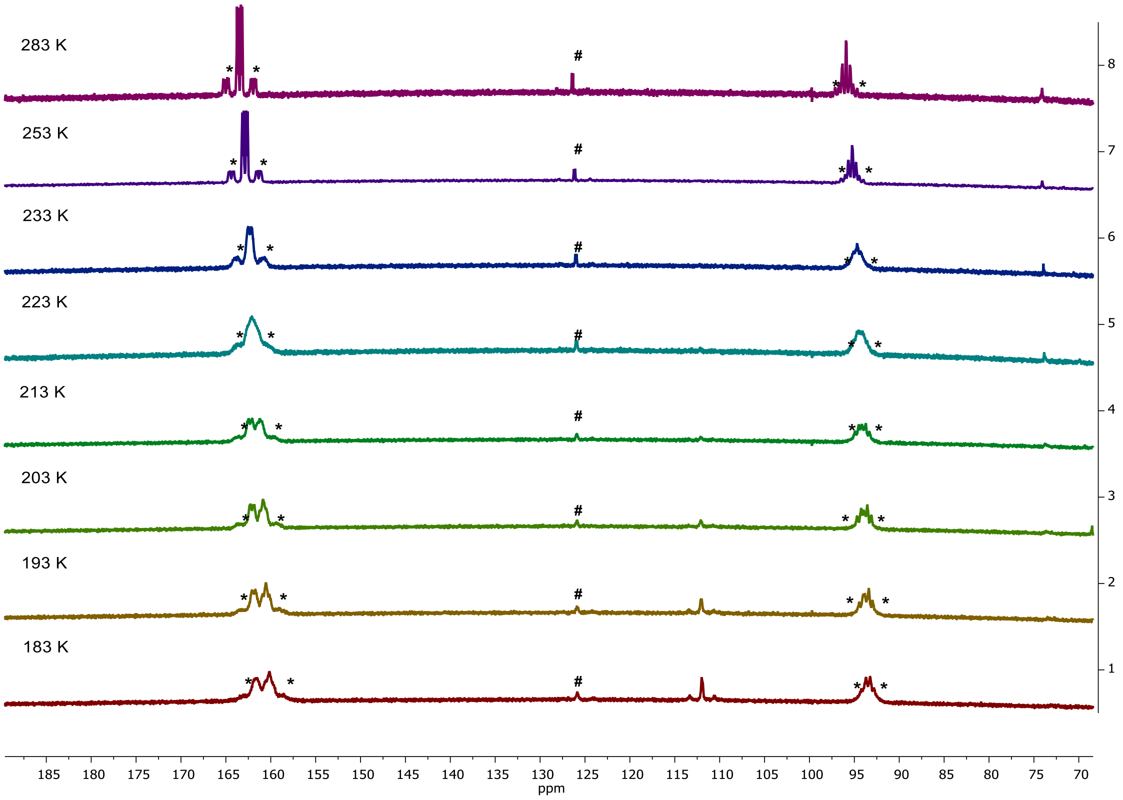

Figure S17. Variable-temperature ^{19}F NMR spectra of *trans*-[Pt(F)(SOF$_3$)(PCy$_3$)$_2$] (**6**), * ^{195}Pt satellites, # *trans*-[Pt(F)(SO$_2$F)(PCy$_3$)$_2$] (**4**), (CD$_2$Cl$_2$, 282 MHz).

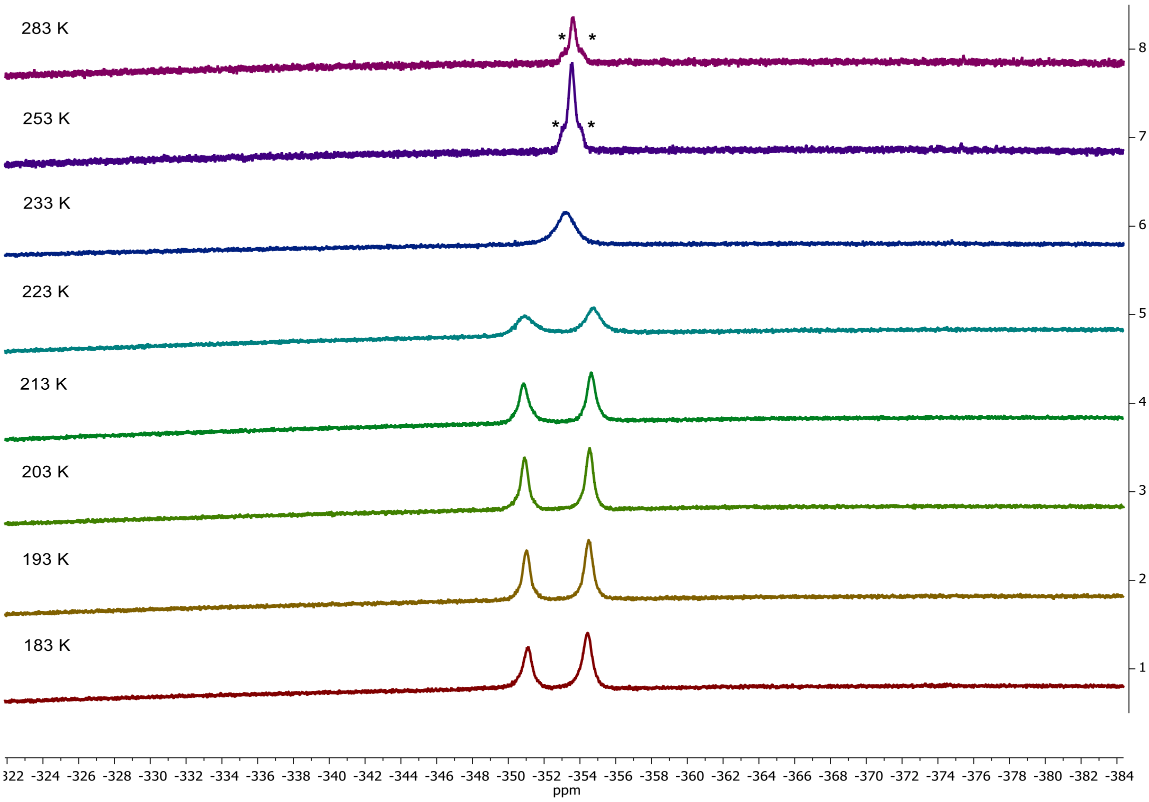

Figure S18. Variable-temperature ^{19}F NMR spectra of *trans*-[Pt(F)(SOF$_3$)(PCy$_3$)$_2$] (**6**), * ^{195}Pt satellites, (CD$_2$Cl$_2$, 282 MHz).

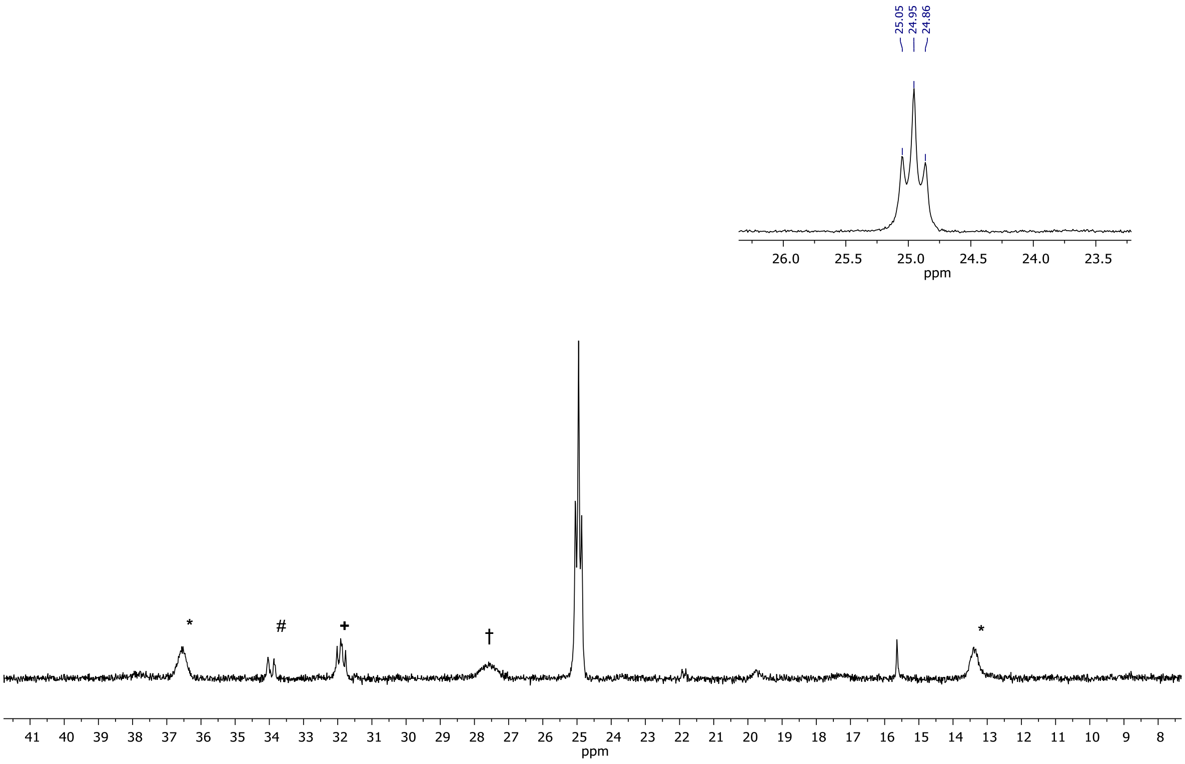

Figure S19. ^{31}P{^{1}H} NMR spectrum of *trans*-[Pt(F)$_2$(PCy$_3$)$_2$] (**7**) at 283 K, * ^{195}Pt satellites, + *trans*-[Pt(F)(SOF)(PCy$_3$)$_2$] (**2**), # *trans*-[Pt(F)(SO$_2$F)(PCy$_3$)$_2$] (**4**), † *trans*-[Pt(F)(SOF$_3$)(PCy$_3$)$_2$] (**6**) (CD$_2$Cl$_2$, 121.5 MHz).

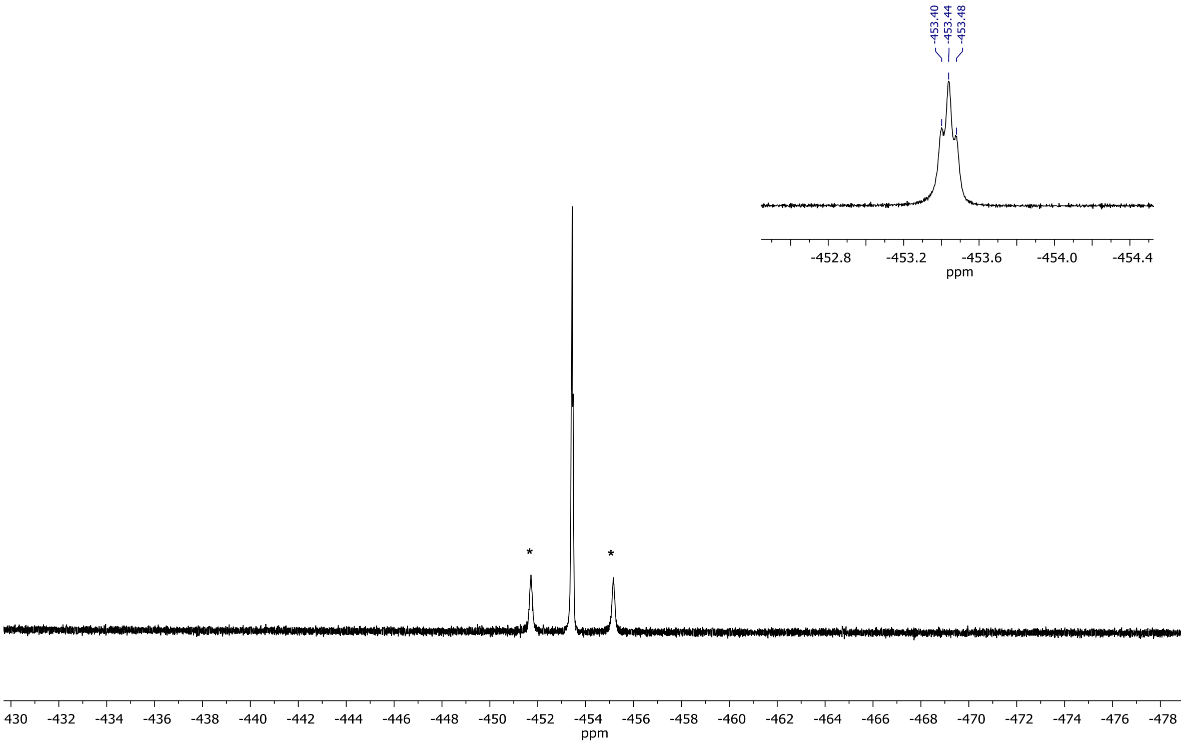

Figure S20. ^{19}F NMR spectrum of *trans*-[Pt(F)$_2$(PCy$_3$)$_2$] (**7**) at 283 K, * ^{195}Pt satellites (CD_2Cl_2, 282 MHz).

10. Structures and Crystallographic Data

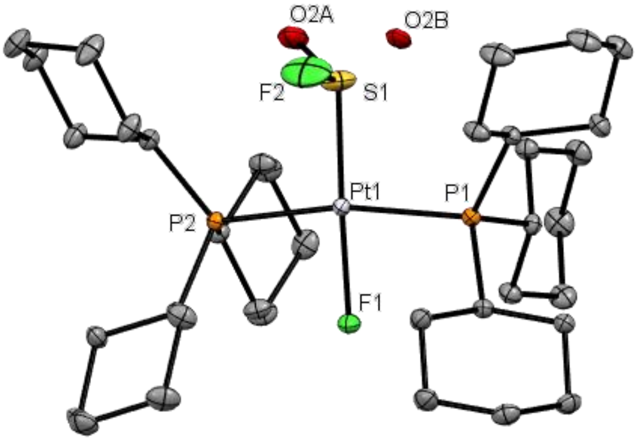

Figure S21. Structure of **2**. Thermal ellipsoids are drawn at 50% probability level. Carbon-bound hydrogen atoms were omitted for clarity. Selected distances [Å] and angles [°]: Pt1–S1 2.1958(7), Pt1–F1 2.047(1), Pt1–P1 2.3359(5), Pt1–P2 2.3383(6), S1–F2 1.637(2), S1–O2A 1.407(2); F1-Pt1-S1 177.26(4), F1-Pt1-P1 84.38(4).

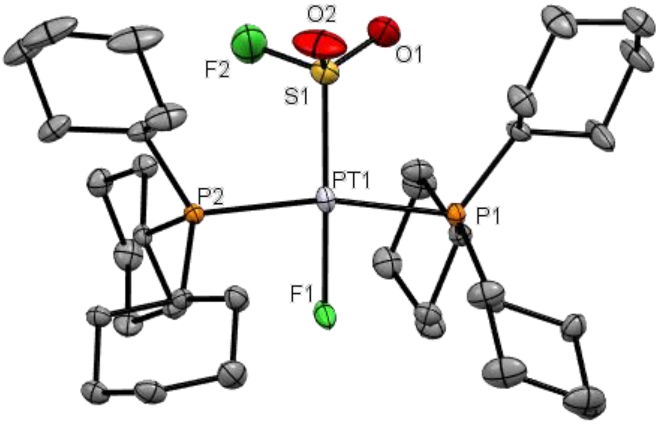

Figure S22. Structure of **4**. Thermal ellipsoids are drawn at 50% probability level. Carbon-bound hydrogen atoms were omitted for clarity. Selected bond lengths [Å] and angles [°]: Pt1–S1 2.221(1), Pt1–F1 2.007(2), Pt1–P1 2.353(1), Pt1–P2 2.349(1), S1–F2 1.549(3), S1–O1 1.436(5), S1–O2 1.496(4); F1-Pt1-S1 179.24(17), F1-Pt1-P1 83.39(3).

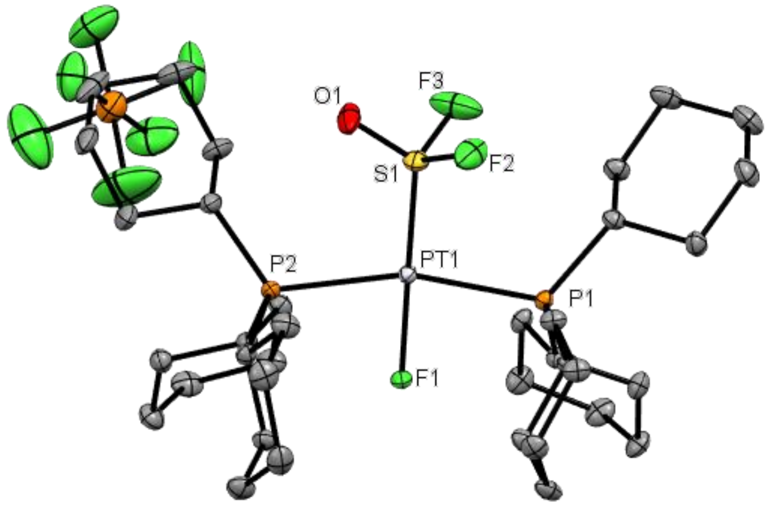

Figure S23. Structure of **5b**. Thermal ellipsoids are drawn at 50% probability level. Carbon-bound hydrogen atoms were omitted for clarity. Selected bond lengths [Å] and angles [°]: Pt1–S1 2.1082(7), Pt1–F1 1.952(1), Pt1–P1 2.4001(6), Pt1–P2 2.3973(7), S1–F2 1.543(2), S1–F2 1.516(2), S1–O1 1.432(2); F1–Pt1–S1 178.04(5), F1–Pt1–P1 82.45(4).

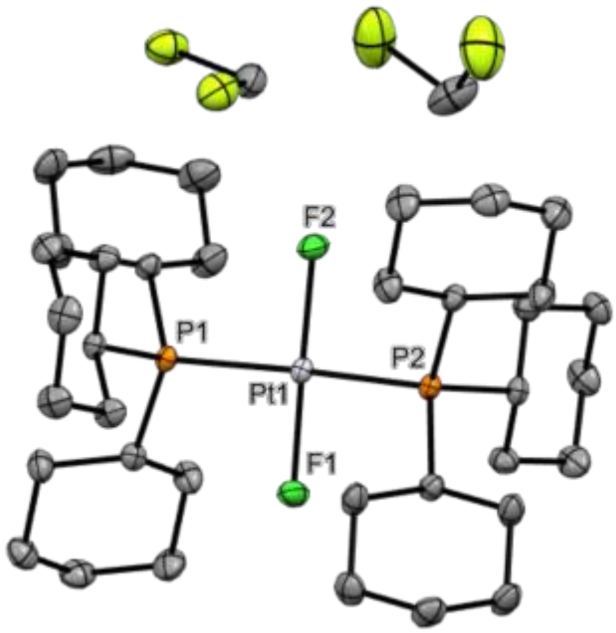

Figure S24. Structure of **7 · 2 CH_2Cl_2**. Thermal ellipsoids are drawn at 50% probability level. Carbon-bound hydrogen atoms were omitted for clarity. Selected bond lengths [Å] and angles [°]: Pt1–F1 1.984(2), Pt1–F2 1.993(2), Pt1–P1 2.3165(6), Pt1–P2 2.3091(6), F1–Pt1–F2 178.14(7), F1–Pt1–P1 89.65(5).

Table S1. Crystallographic data for the complexes **2**, **4**, **5b** and **7**• 2(CH_2Cl_2).

Compound	[Pt(F)(SOF)(PCy_3)$_2$] (**2**)	[Pt(F)(SO_2F)(PCy_3)$_2$] (**4**)
Empirical formula	C_{36} H_{66} F_2 O P_2 Pt S	C_{36} H_{66} F_2 O_2 P_2 Pt S
Formula weight	841.97	857.97
Temperature	100(2) K	100(2) K
Wavelength	0.71073 Å	0.71073 Å
Crystal system	monoclinic	monoclinic
Space group	P 2_1/n	P 2_1/c
a [Å]	13.1036(10)	13.0359(10)
b [Å]	12.6151(9)	12.6836(8)
c [Å]	23.296(2)	23.2891(18)
β [°]	106.326(3)	105.762(3)
Volume [Å^3]	3695.7(5)	3705.9
Z	4	4
Density (calculated)	1.513 Mg/m^3	1.538 Mg/m^3
Absorption coefficient	3.980 mm^{-1}	3.969 mm^{-1}
F(000)	1728	1760
Crystal size	0.395 × 0.154 × 0.059 mm^3	0.233x0.072x0.069 mm^3
Theta range for data collection	2.069x25.242	2.29x24.96
Index ranges	-17<=h<=17, -17<=k<=17, -31<=l<=31	-15<=h<=15, -15<=k<=15, -27<=l<=27
Reflections collected	93669	103967
Independent reflections	9548 [R(int) = 0.0440]	9984 [R(int) = 0.0471]
Completeness to theta = 25.242°	99.9%	99.8%
Absorption correction	Multi-scan	Multi-scan
Max. and min. transmission	0.4923 and 0.2735	0.4899 and 0.3209
Refinement method	Full-matrix least-squares on F^2	Full-matrix least-squares on F^2
Data / restraints / parameters	9548 / 0 / 398	6591 / 0 / 397
Goodness-of-fit on F^2	1.032	1.046
Final R indices [I>2sigma(I)]	R1 = 0.0187, wR2 = 0.0408	R1 = 0.0258, wR2 = 0.0579
R indices (all data)	R1 = 0.0223, wR2 = 0.0419	R1 = 0.0281, wR2 = 0.0590
Extinction coefficient	n/a	n/a
Largest diff. peak and hole	1.239 and -0.806 e.Å^{-3}	1.934 and -1.870 e.Å^{-3}

Compound	$[Pt(F)(SOF_2)(PCy_3)_2][PF_6]$ (**5b**)	$[Pt(F)_2(PCy_3)_2]$ • $2(CH_2Cl_2)$ (**7•** $2(CH_2Cl_2)$)
Empirical formula	C_{36} H_{66} F_9 O P_3 Pt S	C_{38} H_{70} Cl_4 F_2 P_2 Pt
Formula weight	1005.94	963.77
Temperature	100(2) K	100(2) K
Wavelength	0.71073 Å	0.71073 Å
Crystal system	monoclinic	monoclinic
Space group	P 2_1/n	P 2_1/c
a [Å]	23.1588(14)	12.2418(7)
b [Å]	16.0107(9)	17.4422(8)
c [Å]	23.4168(15)	20.4404(12)
β [°]	108.075(2)	103.711(2)
Volume [$Å^3$]	8254.	4240.1(4)
Z	4	4
Density (calculated)	1.619 Mg/m^3	1.510 Mg/m^3
Absorption coefficient	3.636 mm^{-1}	3.671 mm^{-1}
F(000)	4080	1968
Crystal size	0.354x0.290x0.075 mm^3	0.356x0.164x0.081 mm^3
Theta range for data collection	2.20x25.08	2.12x25.05
Index ranges	-27<=h<=27, -19<=k<=19, -27<=l<=27	-14<=h<=14, -20<=k<=20, -24<=l<=24
Reflections collected	417142	241959
Independent reflections	9877 [R(int) = 0.0528]	9384 [R(int) = 0.0371]
Completeness to theta = 25.242°	99.6%	99.9 %
Absorption correction	Multi-scan	Multi-scan
Max. and min. transmission	0.4899 and 0.2064	0.4899 and 0.3380
Refinement method	Full-matrix least-squares on F^2	Full-matrix least-squares on F^2
Data / restraints / parameters	14653/ 0 / 919	7501/ 0 / 424
Goodness-of-fit on F^2	1.044	1.051
Final R indices [I>2sigma(I)]	R1 = 0.0170, wR2 = 0.0406	R1 = 0.0186, wR2 = 0.0490
R indices (all data)	R1 = 0.0189, wR2 = 0.0414	R1 = 0.0202, wR2 = 0.0499
Extinction coefficient	n/a	n/a
Largest diff. peak and hole	0.918 and -0.620 e.$Å^{-3}$	0.883 and -0.969 e.$Å^{-3}$

11. Computational Details

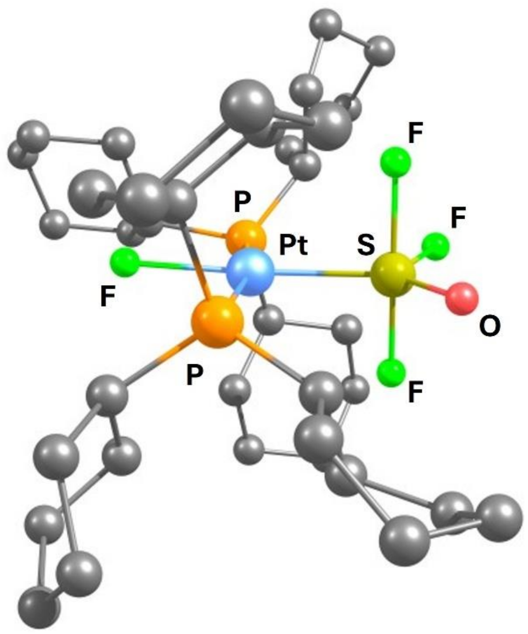

Figure S25. DFT-optimized structure of the cationic part of **6**; all hydrogen atoms as well as the cyclohexyl groups at the phosphorus atoms have been omitted for clarity. B3LYP/cc-pvdz with Grimme D3 dispersion correction including Becke-Johnson damping (RECP with corresponding cc-pvdz basis set for Pt).

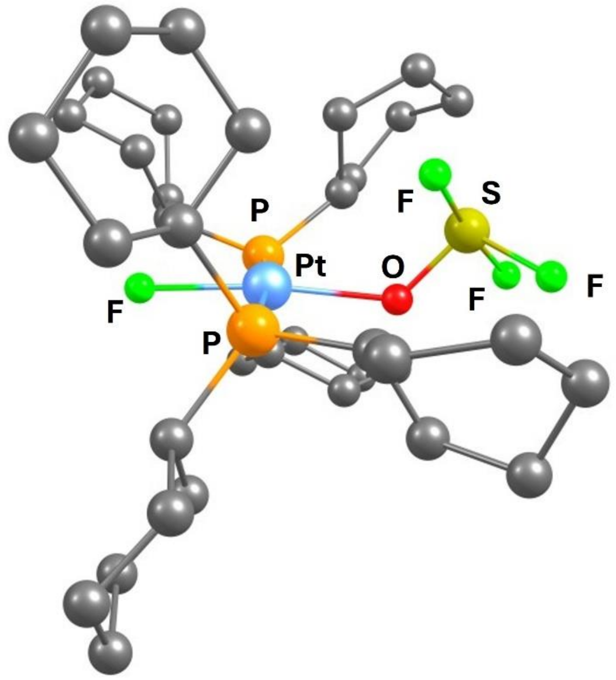

Figure S26: DFT-optimized structure of the cationic part of **6’**; all hydrogen atoms as well as the cyclohexyl groups at the phosphorus atoms have been omitted for clarity. B3LYP/cc-pvdz with Grimme D3 dispersion correction including Becke-Johnson damping (RECP with corresponding cc-pvdz basis set for Pt).

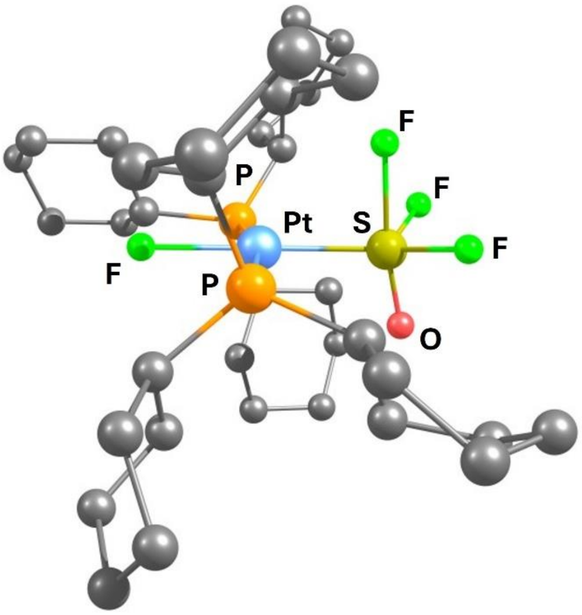

Figure S27. DFT-optimized structure of the cationic part of **6''**; all hydrogen atoms as well as the cyclohexyl groups at the phosphorus atoms have been omitted for clarity. B3LYP/cc-pvdz with Grimme D3 dispersion correction including Becke-Johnson damping (RECP with corresponding cc-pvdz basis set for Pt).

Cartesian coordinates for compound **6**.

Sum of electronic and zero-point energies (Hartree): -3086.089693

Pt	0.07890	-0.16276	0.09537	C	-2.36120	-1.66594	1.73791
F	0.13644	0.42784	2.00378	C	-3.50400	-1.78432	2.76138
P	-2.26357	-0.18410	0.58338	C	-4.79432	-2.38138	2.19566
P	2.44338	0.19168	0.05243	C	-4.52954	-3.71126	1.47666
C	-2.57731	1.30466	1.67008	C	-3.43079	-3.58244	0.41412
C	-1.89879	2.57752	1.10657	C	-2.14066	-3.00006	0.99893
C	-2.40889	3.81354	1.85129	C	3.49992	-1.20377	-0.56428
C	-3.90953	4.05651	1.58126	C	3.06349	-2.58512	-0.00378
C	-4.66757	2.73991	1.31223	C	4.22841	-3.59230	-0.02615
C	-4.05051	1.57176	2.08608	C	5.09271	-3.38894	-1.27368
C	2.93368	0.67056	1.79476	C	5.79078	-2.00937	-1.24292
C	2.89418	-0.53023	2.75668	C	5.01101	-0.98142	-0.39672
C	3.03843	-0.06007	4.20810	C	-3.37673	-0.19622	-0.91400
C	4.32207	0.75141	4.40599	C	-3.07079	1.02288	-1.81279
C	4.38427	1.92820	3.42737	C	-3.58132	0.77032	-3.24562
C	4.23634	1.46928	1.96870	C	-4.86308	-0.08716	-3.25646

C	-5.67342	0.15233	-1.98057
C	-4.90680	-0.34416	-0.73341
C	2.88398	1.68833	-0.97683
C	3.06709	1.41059	-2.47832
C	3.41316	2.70628	-3.22419
C	2.34973	3.78482	-2.99458
C	2.18132	4.07136	-1.49926
C	1.83604	2.79126	-0.73151
H	3.85535	2.03506	-0.58624
H	2.14406	0.98985	-2.89441
H	3.86788	0.67141	-2.63494
H	3.51761	2.49173	-4.30035
H	4.39511	3.08318	-2.87980
H	1.38667	3.43219	-3.40540
H	2.61395	4.70823	-3.53615
H	1.39388	4.82540	-1.33444
H	3.11920	4.50128	-1.09976
H	0.85613	2.41500	-1.06657
H	1.74506	3.00284	0.34672
H	-3.00372	-1.07918	-1.45269
H	-1.99776	1.24424	-1.84680
H	-3.56914	1.90696	-1.38572
H	-2.78818	0.27498	-3.82210
H	-3.77564	1.74502	-3.72392
H	-4.59927	-1.15759	-3.31836
H	-5.46677	0.13290	-4.15107
H	-6.65296	-0.35002	-2.02683
H	-5.88490	1.23200	-1.88614
H	-5.14332	-1.40405	-0.56761
H	-5.26847	0.19926	0.14829
H	-2.00373	1.02088	2.56371
H	-2.09692	2.69452	0.03090
H	-0.81351	2.46588	1.21872
H	-1.82140	4.70025	1.56518
H	-2.23721	3.66799	2.93250
H	-4.03291	4.72791	0.71530
H	-4.35763	4.57872	2.44311
H	-4.64476	2.50821	0.23552
H	-5.73172	2.84826	1.57622
H	-4.67210	0.67310	1.98638
H	-4.06217	1.81794	3.16205
H	-1.44355	-1.43678	2.30537
H	-3.69635	-0.82198	3.25685
H	-3.12966	-2.45898	3.55375
H	-5.26086	-1.66958	1.49781
H	-5.51908	-2.53040	3.01338
H	-5.46071	-4.09019	1.02364
H	-4.21586	-4.46395	2.22397
H	-3.78334	-2.94739	-0.41512
H	-3.21668	-4.56696	-0.03212
H	-1.37122	-2.89748	0.22117
H	-1.73785	-3.71351	1.74118
H	2.09242	1.32040	2.08199
H	3.71826	-1.22518	2.51968
H	1.94784	-1.07268	2.63117
H	3.02324	-0.92951	4.88603
H	2.16168	0.56055	4.46601
H	5.19669	0.09468	4.23940
H	4.39521	1.11416	5.44469
H	5.33004	2.48229	3.54750
H	3.56952	2.63790	3.66143
H	5.10624	0.85261	1.69033
H	4.24890	2.35313	1.31419
H	3.27862	-1.20114	-1.64148
H	2.23096	-2.95152	-0.61620
H	2.67202	-2.49683	1.01825

H	5.30388	0.03946	-0.68565	H	6.80989	-2.10528	-0.83270
H	5.27677	-1.09125	0.66608	S	-0.08691	-0.99691	-1.99541
H	3.82561	-4.61680	0.00856	F	0.20883	-2.62276	-1.35278
H	4.85723	-3.47739	0.87516	F	0.00202	0.63959	-2.70075
H	4.44240	-3.46096	-2.16289	O	-1.26017	-1.40498	-2.78775
H	5.84085	-4.19121	-1.37232	F	1.32651	-1.25655	-2.83939
H	5.90399	-1.62840	-2.27159				

Optimized cartesian coordinates for compound **6'**.

Sum of electronic and zero-point energies (Hartree): -3086.084757

Pt	0.07075	0.26495	0.22921	C	3.91484	-3.57803	0.45190
F	0.27447	2.16924	0.74962	C	4.16197	-3.75811	-1.04832
P	-2.28168	0.41964	0.51086	C	4.96563	-2.56697	-1.61458
P	2.41579	0.33403	0.10922	C	4.67337	-1.25097	-0.85606
C	-2.85039	2.19702	0.47503	C	-3.16412	-0.63167	-0.76195
C	-2.22671	2.97848	-0.70784	C	-2.74307	-0.19385	-2.18402
C	-2.89346	4.35115	-0.82945	C	-3.06282	-1.28722	-3.23376
C	-4.38121	4.22453	-1.22691	C	-4.17152	-2.23668	-2.75309
C	-4.98181	2.88412	-0.76162	C	-5.24578	-1.43548	-2.01391
C	-4.38423	2.44026	0.57713	C	-4.69520	-0.82028	-0.70262
C	3.09292	1.01167	1.71368	C	2.76404	1.64255	-1.17437
C	2.25311	0.58000	2.93160	C	4.12202	2.36190	-1.13242
C	2.74495	1.27393	4.20711	C	4.15086	3.50016	-2.16335
C	4.23856	1.03220	4.44851	C	3.82708	2.99711	-3.57433
C	5.06763	1.45606	3.23186	C	2.48103	2.26532	-3.60491
C	4.58891	0.74964	1.95663	C	2.44378	1.12164	-2.58598
C	-2.48776	-0.14094	2.30159	H	1.99139	2.38442	-0.90339
C	-3.73703	0.24549	3.11308	H	4.93947	1.65451	-1.35053
C	-4.93688	-0.67715	2.89052	H	4.31339	2.77217	-0.12938
C	-4.55901	-2.14828	3.11181	H	5.13611	3.99517	-2.14559
C	-3.34319	-2.56567	2.27550	H	3.40733	4.26438	-1.87097
C	-2.14783	-1.63425	2.49746	H	4.62368	2.30360	-3.90356
C	3.21532	-1.26668	-0.37130	H	3.82748	3.83742	-4.28836
C	3.01954	-2.34711	0.72076	H	2.27726	1.87378	-4.61512

H	1.67062	2.98072	-3.37226
H	3.18472	0.35600	-2.87574
H	1.46809	0.61616	-2.59424
H	-2.71114	-1.61337	-0.56168
H	-1.67373	0.06586	-2.21597
H	-3.29040	0.72758	-2.43562
H	-2.15419	-1.85815	-3.47492
H	-3.37639	-0.79505	-4.16954
H	-3.74365	-3.00027	-2.08114
H	-4.60533	-2.77842	-3.60833
H	-6.12232	-2.06135	-1.78371
H	-5.60625	-0.63243	-2.68127
H	-4.93552	-1.48730	0.13432
H	-5.20761	0.12989	-0.50688
H	-2.37050	2.58395	1.38953
H	-2.35447	2.43119	-1.65394
H	-1.14928	3.06629	-0.52873
H	-2.35290	4.97319	-1.56013
H	-2.80320	4.87165	0.14045
H	-4.49180	4.30998	-2.32073
H	-4.95385	5.06113	-0.79244
H	-4.78774	2.10534	-1.51781
H	-6.07802	2.96038	-0.67992
H	-4.91243	1.55626	0.95565
H	-4.56444	3.23472	1.32188
H	-1.64664	0.44210	2.71560
H	-4.00963	1.29764	2.94896
H	-3.44210	0.17492	4.17680
H	-5.32704	-0.54160	1.86985
H	-5.75395	-0.38845	3.57258
H	-5.42039	-2.79991	2.88946
H	-4.32429	-2.29522	4.18272
H	-3.60266	-2.58857	1.20602
H	-3.04595	-3.59703	2.52391
H	-1.31379	-1.93575	1.85085
H	-1.80236	-1.74704	3.54145
H	2.93835	2.09812	1.59041
H	2.31485	-0.51477	3.05825
H	1.19678	0.82199	2.74938
H	2.15398	0.92853	5.07156
H	2.56241	2.36055	4.11454
H	4.40640	-0.04378	4.64263
H	4.57587	1.57083	5.34955
H	6.13656	1.24259	3.39830
H	4.98125	2.54982	3.09337
H	4.75457	-0.33499	2.06607
H	5.19068	1.07318	1.09550
H	2.58822	-1.54285	-1.23237
H	1.96245	-2.63975	0.73579
H	3.25339	-1.94291	1.71746
H	4.87331	-0.39511	-1.51412
H	5.35208	-1.14861	0.00418
H	3.44408	-4.47579	0.88213
H	4.88593	-3.46023	0.96551
H	3.18748	-3.83376	-1.55678
H	4.69393	-4.70211	-1.24699
H	4.72334	-2.43714	-2.68241
H	6.04724	-2.77691	-1.56105
O	-0.09679	-1.77151	-0.08114
S	-0.40050	-2.52884	-1.39178
F	0.81268	-1.61915	-2.35984
F	0.69505	-3.76571	-1.32823
F	-1.53301	-3.59418	-0.55278

Optimized cartesian coordinates for the **6’’**.

Sum of electronic and zero-point energies (Hartree): -3086.056067

Pt	-0.08858	0.08545	0.08560
F	-0.13774	-0.42824	2.02095
P	2.24885	0.15675	0.58580
P	-2.45526	-0.20029	0.07848
C	2.59206	-1.32702	1.66947
C	1.93585	-2.61021	1.10385
C	2.46030	-3.83699	1.85386
C	3.96601	-4.05736	1.59196
C	4.70695	-2.73021	1.32595
C	4.06762	-1.56923	2.09231
C	-2.94895	-0.64031	1.82860
C	-2.86037	0.57102	2.77451
C	-3.02810	0.12740	4.23161
C	-4.34706	-0.62299	4.43726
C	-4.45899	-1.80951	3.47502
C	-4.28511	-1.37969	2.01038
C	5.68937	-0.05056	-1.95664
C	4.89298	0.41087	-0.71419
C	-2.97496	-1.67420	-0.94506
C	-3.17507	-1.38297	-2.44226
C	-3.58149	-2.66181	-3.18686
C	-2.55727	-3.78187	-2.97877
C	-2.37235	-4.07977	-1.48752
C	-1.96467	-2.81639	-0.72294
H	-3.95177	-1.98606	-0.53926
H	-2.24699	-0.99342	-2.87942
H	-3.95067	-0.61425	-2.58108
H	-3.69562	-2.43890	-4.26023
H	-4.57137	-3.00123	-2.82678
H	-1.58888	-3.46741	-3.40749
H	-2.86855	-4.69236	-3.51715
C	2.30237	1.63386	1.74943
C	3.43341	1.76538	2.78457
C	4.71348	2.40273	2.24010
C	4.42173	3.73559	1.53716
C	3.33624	3.59416	0.46268
C	2.05480	2.96981	1.02266
C	-3.42158	1.25343	-0.54213
C	-2.87025	2.60520	-0.01498
C	-3.93549	3.71494	-0.11415
C	-4.81554	3.51215	-1.35071
C	-5.64651	2.21469	-1.22784
C	-4.94266	1.15265	-0.35464
C	3.37046	0.20787	-0.90663
C	3.12452	-1.02618	-1.80312
C	3.63526	-0.76011	-3.23374
C	4.87806	0.15280	-3.23812
H	1.88830	-4.73342	1.56673
H	2.28096	-3.69161	2.93383
H	4.10386	-4.72771	0.72736
H	4.41700	-4.57231	2.45664
H	4.68998	-2.50110	0.24848
H	5.77037	-2.82282	1.59867
H	4.67515	-0.66105	1.99171
H	4.07903	-1.81115	3.16923
H	1.38627	1.37586	2.30646
H	3.64567	0.80195	3.26972
H	3.03470	2.41978	3.58200
H	5.20484	1.71282	1.53721
H	5.42645	2.55821	3.06702
H	5.34756	4.14468	1.09934
H	4.08174	4.46895	2.29214

H	-1.61145	-4.86388	-1.33967
H	-3.31838	-4.47483	-1.07160
H	-0.97650	-2.47661	-1.07365
H	-1.86090	-3.03337	0.35315
H	2.97027	1.08072	-1.44321
H	2.06398	-1.30059	-1.84075
H	3.65981	-1.88349	-1.36710
H	2.82796	-0.30694	-3.82532
H	3.87652	-1.72808	-3.70382
H	4.56880	1.21092	-3.30298
H	5.49593	-0.04157	-4.12889
H	6.64565	0.49500	-1.99684
H	5.94823	-1.11970	-1.86146
H	5.09020	1.47676	-0.53806
H	5.26333	-0.12668	0.16779
H	2.01032	-1.05315	2.56116
H	2.14272	-2.72617	0.02946
H	0.84761	-2.51393	1.20747
H	-5.31776	0.14955	-0.60992
H	-5.18691	1.31418	0.70710
H	-3.43815	4.69706	-0.14404
H	-4.57501	3.71945	0.78721
H	-4.16230	3.45764	-2.23887
H	-5.48201	4.37466	-1.50916
H	-5.83468	1.79995	-2.23223
H	3.71365	2.98162	-0.37292
H	3.10133	4.57939	0.02850
H	1.30106	2.85237	0.23095
H	1.62506	3.66231	1.76993
H	-2.13313	-1.32055	2.12024
H	-3.65405	1.29619	2.52539
H	-1.89186	1.07047	2.64431
H	-2.97545	1.00480	4.89725
H	-2.18028	-0.52776	4.50082
H	-5.19099	0.06972	4.25881
H	-4.43894	-0.96774	5.48059
H	-5.42910	-2.31886	3.59885
H	-3.67783	-2.55167	3.72252
H	-5.12537	-0.72899	1.71740
H	-4.33368	-2.27232	1.36955
H	-3.21409	1.21826	-1.62155
H	-1.98297	2.86293	-0.60737
H	-2.52823	2.51685	1.02482
H	-6.63519	2.43489	-0.79166
S	-0.00574	0.67027	-2.08464
F	-0.01981	2.43427	-1.38503
O	0.02989	-0.62115	-2.83661
F	1.30015	1.36795	-2.88945
F	-1.32782	1.31867	-2.89507

12. References

[1] a) T. Yoshida, S. Otsuka, D. Jones, J. Spencer, P. Binger, A. Brinkmann, P. Wedemann, *Inorganic Syntheses: Reagents for Transition Metal Complex and Organometallic Syntheses* **1990**, *28*, 113-119; b) J. M. Ritchey, D. C. Moody, R. Ryan, *Inorg. Chem.* **1983**, *22*, 2276-2280; c) C. Berg, T. Braun, M. Ahrens, P. Wittwer, R. Herrmann, *Angew. Chem. Int. Ed.* **2017**, *56*, 4300-4304.

[2] G. M. Sheldrick, *University of Gottingen* **1996**.

[3] G. M. Sheldrick, *Acta Cryst.A* **2015**, *71*, 3-8.

[4] G. M. Sheldrick, *Acta Cryst. C* **2015**, *71*, 3-8.

[5] G. W. T. M. J. Frisch, H. B. Schlegel, G. E. Scuseria, M. A. Robb, J. R. Cheeseman, G. Scalmani, V. Barone, G. A. Petersson, H. Nakatsuji, M. C. X. Li, A. Marenich, J. Bloino, B. G. Janesko, R. Gomperts, B. Mennucci, H. P. Hratchian, J. V. Ortiz, A. F. Izmaylov, J. L. Sonnenberg, D. Williams-Young, F. Ding, F. Lipparini, F. Egidi, J. Goings, B. Peng, A. Petrone, T. Henderson, D. Ranasinghe, V. G. Zakrzewski, J. Gao, N. Rega, G. Zheng, W. Liang, M. Hada, M. Ehara, K. Toyota, R. Fukuda, J. Hasegawa, M. Ishida, T. Nakajima, Y. Honda, O. Kitao, H. Nakai, T. Vreven, K. Throssell, J. A. M. Jr., J. E. Peralta, F. Ogliaro, M. Bearpark, J. J. Heyd, E. Brothers, K. N. Kudin, V. N. Staroverov, T. Keith, R. Kobayashi, J. Normand, K. Raghavachari, A. Rendell, J. C. Burant, S. S. Iyengar, J. Tomasi, M. Cossi, J. M. Millam, M. Klene, C. Adamo, R. Cammi, J. W. Ochterski, R. L. Martin, K. Morokuma, O. Farkas, J. B. Foresman, D. J. F. Gaussian, *Inc.: Wallingford CT* **2016**.

[6] K. A. Peterson, D. Figgen, M. Dolg, H. Stoll, *J. Chem. Phys.* **2007**, *126*.

[7] T. Lu, F. Chen, *J. Comput. Chem.* **2012**, *33*, 580-592.

[8] S. Grimme, S. Ehrlich, L. Goerigk, *J. Comput. Chem.* **2011**, *32*, 1456-1465.

[9] D. Dirican, M. Talavera, T. Braun, *Chem. Eur. J.* **2021**, *27*, 17707-17712.

[10] P. H. M. Budzelaar, *gNMR*, *Version 4.1*, *Adept Scientific plc*, *Letchworth* **2001**.
